# Identification of a novel inhibitor of liver cancer cell invasion and proliferation through regulation of Akt and Twist1

**DOI:** 10.1038/s41598-021-95933-4

**Published:** 2021-08-18

**Authors:** Jain Ha, Sewoong Lee, Jiyoung Park, Jihye Seo, Eunjeong Kang, Haelim Yoon, Ba Reum Kim, Hyeon Kyu Lee, Seong Eon Ryu, Sayeon Cho

**Affiliations:** 1 Laboratory of Molecular and Pharmacological Cell Biology, College of Pharmacy, Chung-Ang University, Seoul, 06974 Republic of Korea; 2 Korea Chemical Bank, Korea Research Institute of Chemical Technology, Yuseong, P.O. Box 107, Daejeon, 34114 Republic of Korea; 3 Department of Bioengineering, College of Engineering, Hanyang University, Seoul, 04763 Republic of Korea

**Keywords:** Cell biology, Drug discovery

## Abstract

When primary cancer faces limited oxygen and nutrient supply, it undergoes an epithelial–mesenchymal transition, which increases cancer cell motility and invasiveness. The migratory and invasive cancer cells often exert aggressive cancer development or even cancer metastasis. In this study, we investigated a novel compound, 3-acetyl-5,8-dichloro-2-((2,4-dichlorophenyl)amino)quinolin-4(1H)-one (ADQ), that showed significant suppression of wound healing and cellular invasion. This compound also inhibited anchorage-independent cell growth, multicellular tumor spheroid survival/invasion, and metalloprotease activities. The anti-proliferative effects of ADQ were mediated by inhibition of the Akt pathway. In addition, ADQ reduced the expression of mesenchymal markers of cancer cells, which was associated with the suppressed expression of Twist1. In conclusion, ADQ successfully suppressed carcinogenic activity by inhibiting the Akt signaling pathway and Twist1, which suggests that ADQ may be an efficient candidate for cancer drug development.

## Introduction

Liver cancer is the seventh most commonly diagnosed cancer and the third leading cause of death from cancer worldwide in 2018^1^. The primary cancer cells undergo physiological changes, which are mediated by suppression of E-cadherin and upregulation of N-cadherin along with the massive expression of matrix metalloproteinases (MMP) that can degrade the extracellular matrix (ECM)^2^. The different expression pattern of E-cadherin and N-cadherin is called “cadherin switching,” which is a hallmark of molecular alterations associated with cancer development^3^. As a result, primary cancer develops the potential to escape from the primary site to other parts of the body and eventually develops into secondary cancers.

In normal cells, the Akt pathway participates in the regulation of cell proliferation via balancing cell cycle progression and apoptosis^4^. When the Akt pathway is activated, it transduces intracellular signaling that induces cell proliferation^5^. The activities of forkhead box O (FoxO) transcription factors are down-regulated via phosphorylation by Akt^6^. The FoxO family exerts tumor-suppressive functions by inducing the expression of cyclin-dependent kinase inhibitors (CDKI), which ensure strict CDK regulation during the cell cycle process^7^. Therefore, the suppression of FoxO transcription factors induced by hyperactivation of Akt leads to the inhibition of CDKI expression, resulting in continuous cell proliferation. Akt is often found highly phosphorylated in most liver cancer cell lines and liver cancer tissues from patients^8^. Therefore, the proper regulation of the Akt pathway is one of the crucial goals for anti-cancer strategies.

Several transcription factors that function in the development of cancer have been reported in recent years^9,10^. The functions of the Twist family have been extensively studied in physiology and pathology, including organogenesis, cell stemness, senescence, angiogenesis, chemoresistance, and metastasis^11^. As a transcription factor, Twist1 regulates the expression of E-cadherin and N-cadherin that are associated with the progression of cancer^9^. It has been reported that high expression of Twist1 is associated with aggressive cancers such as breast cancer, gastric cancer, pancreatic cancer, and liver cancer^12^. Therefore, the regulatory mechanism of Twist1 needs to be researched to identify proper therapeutic strategies. To identify and confirm the anti-cancer effect of a chemical, 3-acetyl-5,8-dichloro-2-((2,4-dichlorophenyl)amino)quinolin-4(1H)-one (ADQ), on a liver cancer cell line, various cell-based assays were performed in this study. The scaffold structure of ADQ was previously reported to have activity toward several proteases^13^. In this study, the effects of ADQ suppressing cancer cell migration and invasion were evaluated comprehensively using various liver cancer-derived cell lines, including several HCC such as Huh7, Hep3B, and PLC-PRF-5 and a liver endothelial cancer cell line, SK-Hep1.

## Results

### ADQ suppressed the cancerous physiologies of liver cancer cells

**Figure 1:**
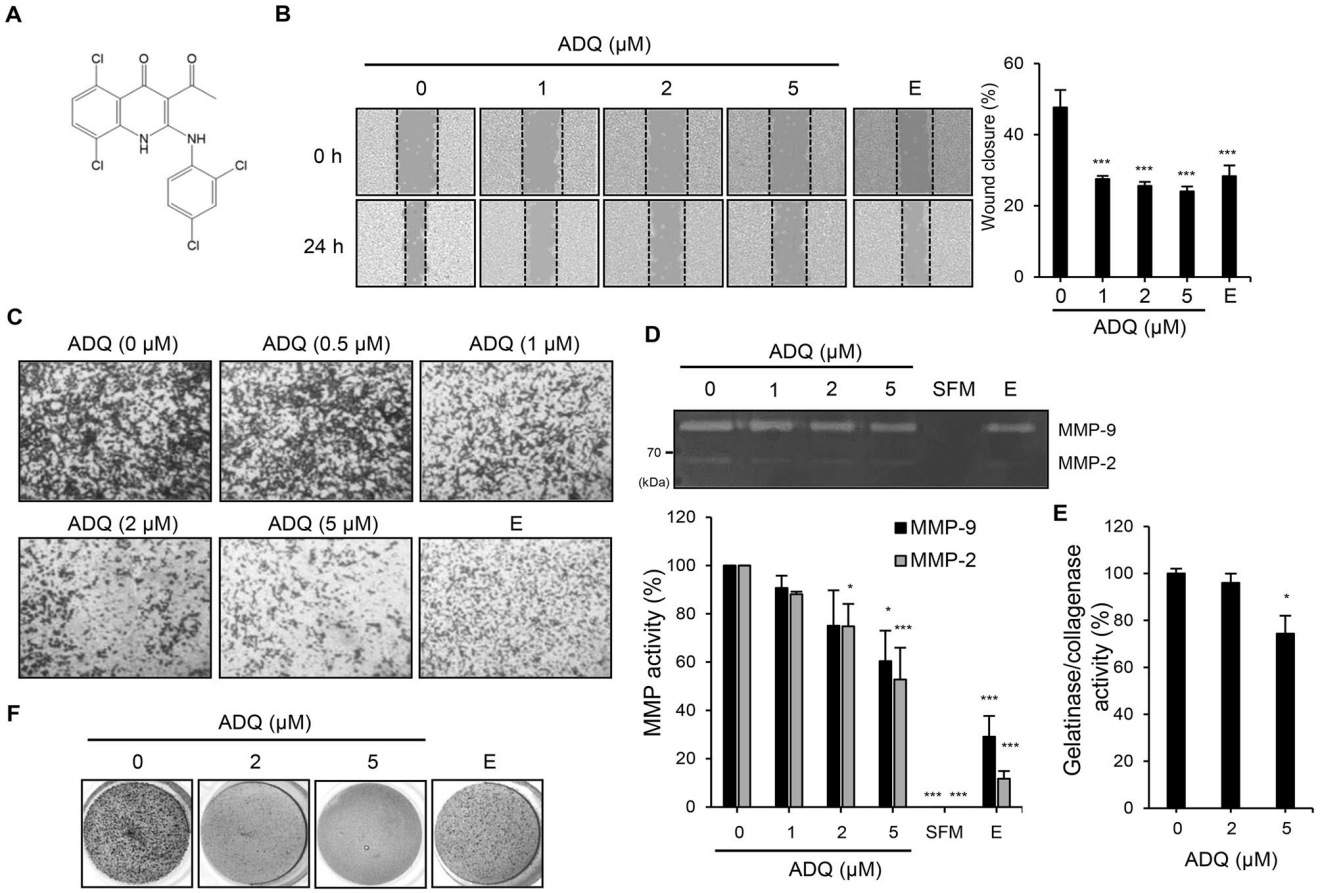
The cancerous characteristics of SK-Hep1 cells were inhibited by ADQ treatment. (**A**) Chemical structure of ADQ. (**B**) The wound was created using SPL Scratcher, and then the cells were incubated with ADQ or emodin (E; 20 μM; positive control) in 1% FBS media. Microscopic images were captured at 0 and 24 h. The wound closure values were quantified by measuring the percent of wound size compared to the 0 h point of each sample (0%), and the relative wound closure is shown as a bar graph. (**C**) SK‑Hep1 cells were treated with ADQ or emodin (E; 20 μM; positive control) in 1% FBS medium. After incubation for 21 h, cells invading the lower surface of the chambers were fixed and stained. (**D**) SK-Hep1 cells were incubated with ADQ or emodin (E; 20 μM; positive control) for 24 h in serum-free medium. The culture media were collected and analyzed by gelatin zymography. Fold values were calculated relative to the ADQ-untreated control. SFM, serum-free media (a negative control). (**E**) The gelatinase/collagenase activity of cells treated with ADQ was accessed using EnzChek Gelatinase/Collagenase Assay Kit. (**F**) SK-Hep1 cells were seeded with 0.3% low-melting agarose and were incubated with ADQ or emodin (E; 40 μM; positive control). After incubation for 14 days, the colonies were stained with 0.5% crystal violet, and images were taken. Data are representative of three experiments and expressed as the means ± SEM. Data were analyzed by one-way ANOVA followed by Holm-Šídák's post hoc test; *p < 0.05, **p < 0.01, and ***p < 0.001 relative to the ADQ-untreated control.

The chemical compound library (~ 3300 compounds) was screened through wound healing assays with SK-Hep1 cells. The compound ADQ (Fig. 1A) was identified as one of the most potent inhibitors among the 3300 compounds screened (Fig. S1A). SK-Hep1 cells treated with 10 μM ADQ showed a notable reduction in cell viability (Fig. S1B). Therefore, ADQ was applied at a maximum concentration of 5 μM in the following assays. The ability of ADQ to suppress the migration of cancer cells was examined using wound healing assays (Fig. 1B). The migratory ability of the cells was significantly decreased by ADQ treatment (1 μM: ***p = 0.0008; 2 μM: ***p = 0.0006; 5 μM: ***p = 0.0005). The anti-migratory effects of ADQ were observed over time (Fig. S1C). The wound closure of the cells treated with the ADQ was only around 50% of the control, which represents significant suppression of cell migration. In addition, the inhibitory effect of ADQ on wound closure was similar to that of emodin (20 μM: ***p = 0.0008), a well-known tumor suppressor compound for various cancer types^14^. The invasion assays were performed using Matrigel to investigate the effect of ADQ (Fig. 1C). SK-Hep1 cells treated with increasing concentrations of ADQ showed less invasion than the ADQ-untreated control. ADQ showed dramatic suppression of cell invasion at a relatively lower concentration than emodin. The invasion of other liver cancer cells, Huh7 and PLC-PRF-5, was also notably inhibited by ADQ (Fig. S1D). Gelatin zymography was performed to evaluate the effect of ADQ on the activities of MMPs (Fig. 1D). The protease activities of MMP-2 and -9 were inhibited by treatment with ADQ. In particular, the MMP-2 activity was almost entirely suppressed by ADQ (2 μM: *p = 0.0416; 5 μM: ***p = 0.0002). The inhibition of MMP activities was also confirmed by MMP enzymatic activity assay (Fig. 1E). Cancer cells that are going through metastatic progression also show anchorage-independent growth^15^. Therefore, a soft-agar colony formation assay was performed to observe the effect of ADQ on the anchorage-independent growth of SK-Hep1 cells (Fig. 1F). During colony formation in the 0.3% soft agar, ADQ showed notable inhibition of colony formation. These data indicate that ADQ inhibits the cancerous physiologies of SK-Hep1 cells, including cell migration, invasion, anchorage-independent growth, and MMP activities.

### Invasion of multicellular tumor spheroid (MTS) was inhibited by ADQ

**Figure 2:**
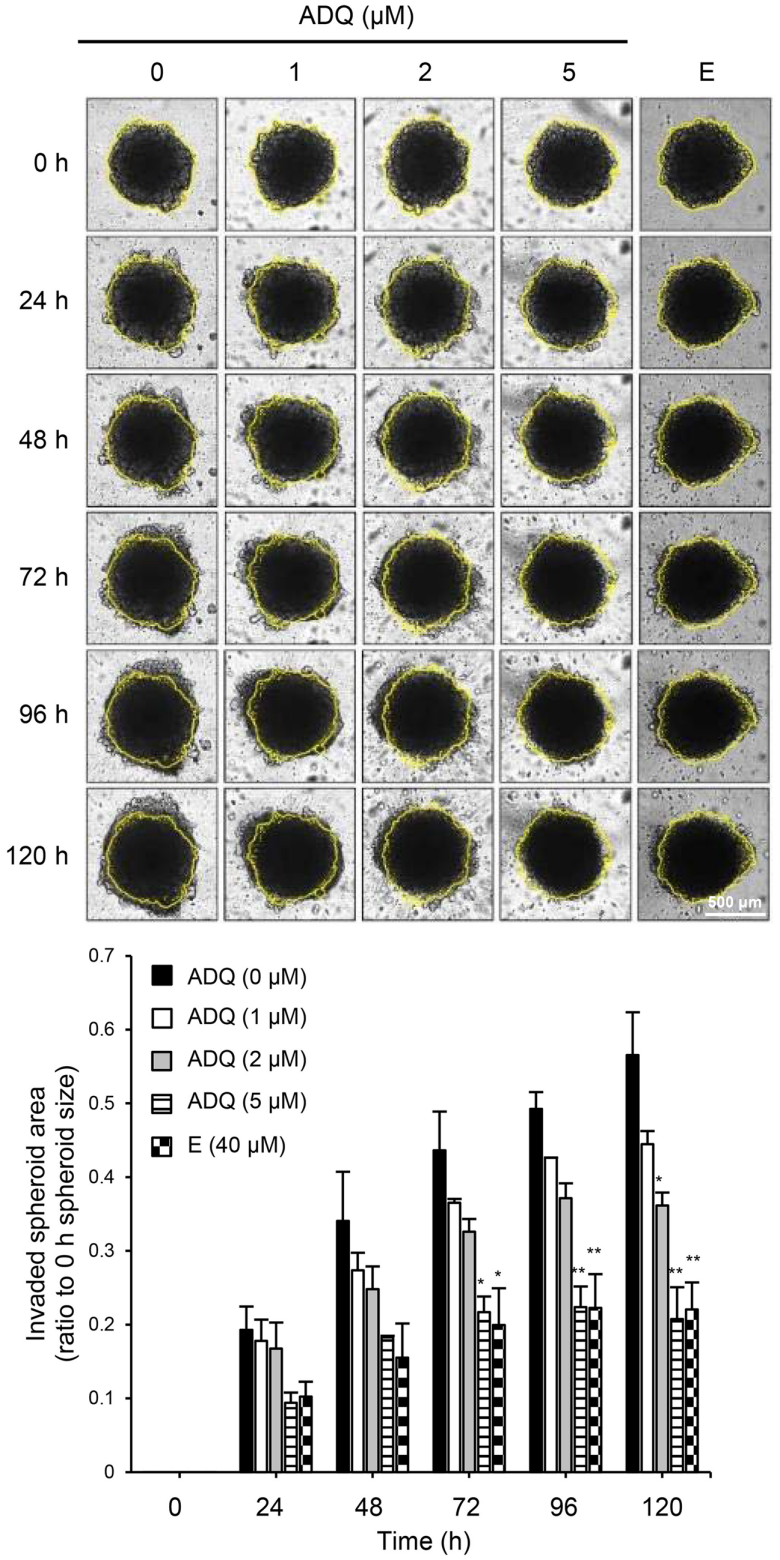
MTS invasion in Matrigel was inhibited by treatment with ADQ. SK-Hep1 spheroids were incubated with ADQ or emodin (E; 40 μM; positive control) for 48 h in 10% FBS media. Microscopic images were captured by a JuLI stage real-time imaging system at each time point. Yellow lines indicate the outline of the MTS at 0 h. The invading area or length of the spheroids was measured using ImageJ. The measured value of each time point was normalized to that of 0 h. The scale bar is 500 μm. Data are representative of three experiments and expressed as the means ± SEM. Data were analyzed by one-way ANOVA followed by Holm-Šídák's post hoc test; *p < 0.05, **p < 0.01, and ***p < 0.001 relative to the ADQ-untreated control.

MTS growth or invasion assays have been utilized to study cancer cell growth and screen anti-cancer drugs because this three-dimensional (3D) culture system mimics the microenvironment of cancer cells that grow in multi-layers and invade into adjacent tissues^16–18^. In our study, an MTS invasion assay was performed to identify the effect of ADQ in conditions similar to the in vivo cancer microenvironment. In comparison to ADQ-untreated MTS, which invaded into the surrounding Matrigel, MTS treated with ADQ showed lesser invasion (Fig. 2). Comparing the yellow outline of MTS observed at the 0 h-time point to the spheroids at each subsequent time point, the MTS invasions were dose-dependently suppressed by ADQ treatment. When the MTS was incubated with 5 μM ADQ, the invasion of MTS was almost completely repressed, which was a more substantial effect than 40 μM emodin treatment. These results suggest that ADQ has the potential to inhibit the invasion of cancer cells in vivo.

### ADQ showed anti-cancer effects via the Akt pathway

**Figure 3:**
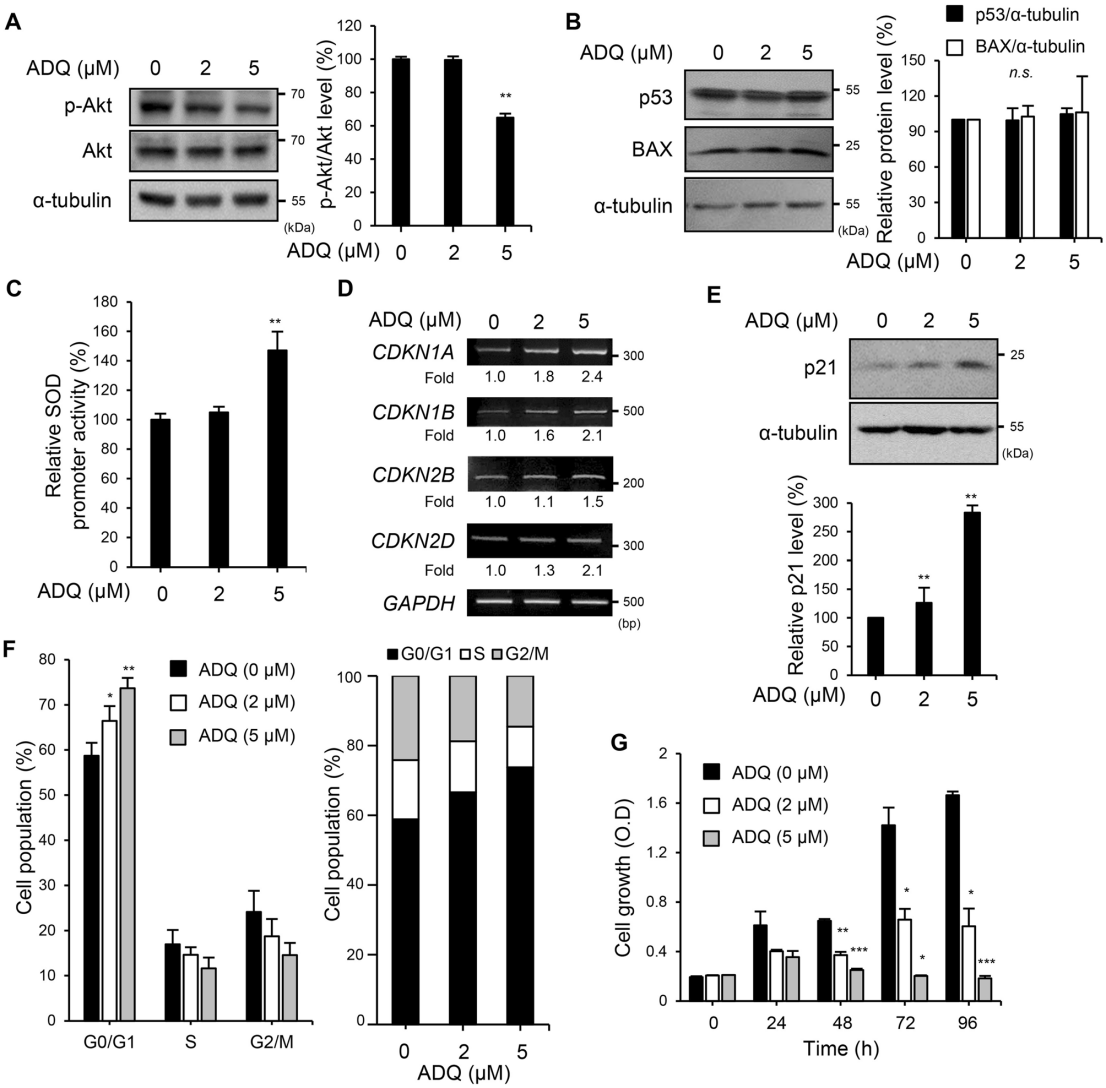
The Akt signaling pathway and its downstream effectors were altered by ADQ. SK-Hep1 cells were treated with ADQ for 24 h in 10% FBS-containing media. (**A**) The expression levels of p-Akt (Ser473), Akt, and α-tubulin were detected by specific antibodies. (**B**) The expression levels of p53, BAX, and α-tubulin were detected by specific antibodies. (**C**) SK-Hep1 cells were co-transfected with the pSOD-Luc reporter and gWIZ-GFP. The cells were treated with the indicated concentrations of ADQ for 24 h in culture media containing 10% FBS. The luciferase activities were normalized against the GFP levels. (**D**) The transcription levels of *CDKN1A*, *CDKN1B*, *CDKN2B*, *CDKN2D*, and *GAPDH* were analyzed by RT-PCR. (**E**) The expression levels of p21 and α-tubulin were detected by specific antibodies. (**F**) The cells were harvested after 24 h of incubation with ADQ in 10% FBS media and analyzed. The bar graph shows the distribution of cells in the different phases of the cell cycle. (**G**) SK-Hep1 cells (1500 cells/well) were seeded in 96-well plates. Cells were treated with the indicated concentration of ADQ in media containing 10% FBS. At each indicated time point, cellular growth was measured using the Ez-Cytox solution. Data are representative of three experiments and expressed as the means ± SEM. Data were analyzed by one-way ANOVA followed by Holm-Šídák's post hoc test; *p < 0.05, **p < 0.01, and ***p < .001 relative to the ADQ-untreated control.

The various intracellular signaling pathways were analyzed to sort out the target pathway of ADQ, and ADQ suppressed the Akt pathway (Fig. 3A). SK-Hep1 cells were treated with LY294002, a PI3K inhibitor, to inhibit the Akt activation specifically. The cells treated with LY294002 showed suppression in wound healing, migration, and invasion as similar to the cells treated with ADQ (Fig. S2). In addition, The Akt pathway has been reported to regulate cell survival^4^. The apoptosis-related proteins were evaluated to investigate whether the suppressed cell growth was due to apoptosis. The levels of the pro-apoptotic protein BAX^19^ were not significantly affected by ADQ treatment (Fig. 3B, P = 0.9468). The levels of p53 that induce apoptosis^19^ were also not altered by ADQ, indicating that the anti-cancer effects of ADQ are not significantly correlated with apoptosis.

The FoxO transcription factor is regulated by Akt, and it induces the expression of CDKIs as it translocates to the nucleus^7^. When ADQ was treated to SK-Hep1 cells, FoxO3a translocated to the nucleus (Fig. S3). The promoter activity of the manganese superoxide dismutase (MnSOD) gene (*sod2*), a FoxO target gene, was evaluated by luciferase reporter assays. The SOD promoter activity was significantly up-regulated by 5 μM ADQ, which suggests that FoxO activity is increased in response to ADQ (Fig. 3C, 5 μM: **p = 0.0033). In addition, the mRNA levels of the CDKI genes that are FoxO target genes were evaluated by RT-PCR after treatment with ADQ. The transcriptional levels of *CDKN1A*, *CDKN1B*, *CDKN2B*, and *CDKN2D* were increased by ADQ (Fig. 3D). The altered transcriptional levels of FoxO target genes were also quantified via qRT-PCR, which all showed significance (Fig. S4, *CDKN1A*: 5 μM: *p = 0.0176; *CDKN1B*: 5 μM: *p = 0.0269; *CDKN2B*: 5 μM: *p = 0.0154; *CDKN2D*: 5 μM: **p = 0.0035). The levels of the p21 protein, which is encoded by the *CDKN1A* gene, were also increased by ADQ (Fig. 3E). To further clarify whether the anti-migratory effects of ADQ were due to the regulation of the Akt/FoxO pathway, p21 proteins were knocked-down and the wound healing assay was performed (Fig. S5). The anti-migratory effects of ADQ were not significantly altered by the p21 knock-down (2 μM: *p* = 0.7512; 5 μM: *p* = 0.8444 at 24 h point), which suggests that the suppression of cell migration by ADQ was not mediated by p21. The cell cycle distribution was evaluated during treatment with ADQ. Cells treated with ADQ showed an increased population in the G0/G1 phase and a decreased population in the G2/M phase (Fig. 3F). When cells were treated with 5 μM ADQ, cell populations in both G0/G1 and the G2/M phase showed significant changes. Cell proliferation was observed to evaluate the anti-cancer effect of ADQ further. Treatment with ADQ successfully suppressed the growth of SK-Hep1 cells (Fig. 3G). Cell growth was almost completely repressed by 5 μM ADQ. Furthermore, the effect of ADQ on cellular growth was also evaluated in 3D culture using MTS (Fig. S6). The MTS growth was measured without Matrigel to observe only the growth. When ADQ was added, the growth of the MTS was suppressed compared to the control. The ADQ treatment did not significantly induce the increase in the apoptotic cell population (Fig. S7, *P* = 0.3431). Taken together, these data suggest that the Akt/FoxO pathway is inhibited by ADQ, which results in cell cycle arrest, not apoptotic cell death.

### The Twist1 transcription factor was down-regulated by ADQ

**Figure 4:**
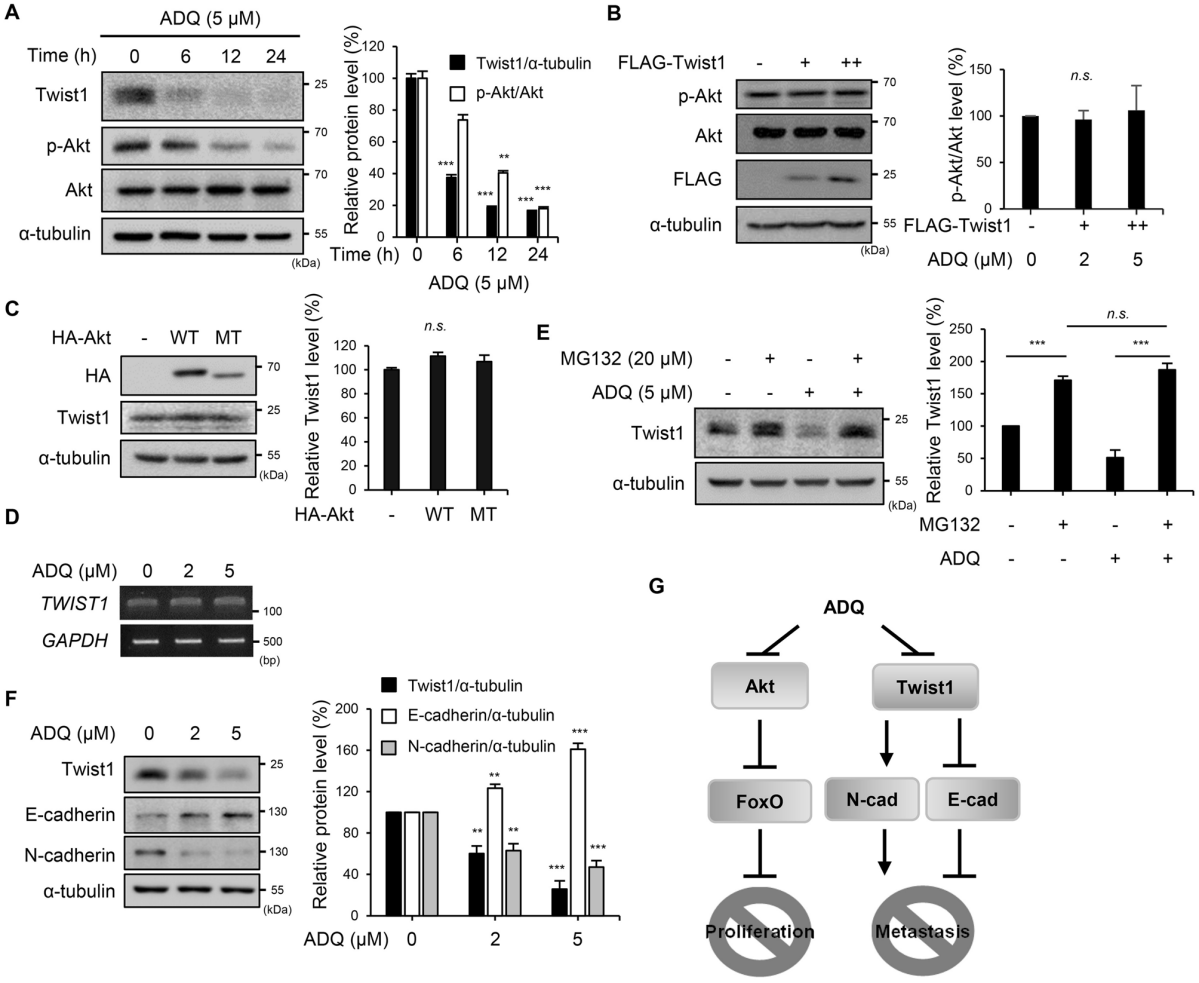
The stability of the Twist1 transcription factor was decreased by ADQ. (**A**) SK-Hep1 cells were treated with 5 μM ADQ in 10% FBS-containing media and were harvested at the indicated time points. The expression levels of Twist1, p-Akt (Ser473), Akt, and α-tubulin were detected by specific antibodies. (**B**) FLAG-Twist1 (0, 2, or 4 μg) was transfected into SK-Hep1 cells. After 48 h, total cell lysates were harvested and separated by SDS-PAGE. The expression levels of FLAG, p-Akt (Ser473), Akt, and α-tubulin were detected by specific antibodies. (**C**) SK-Hep1 cells were transfected for 48 h with HA-AKT WT or MT K179M. The lysates were separated by SDS-PAGE, and the expression levels of HA, Twist1, and α-tubulin were detected by specific antibodies. WT, wild type; MT, mutant type. (**D**) RNA was harvested from the cells treated with ADQ for 24 h in 10% FBS media. The transcription levels of *TWIST1* and *GAPDH* were evaluated by RT-PCR. (**E**) Cells were treated either with ADQ (5 μM) or MG132 (20 μM) for 6 h in 10% FBS media. The expression levels of Twist1 and α-tubulin in the cell lysates were analyzed by specific antibodies. (**F**) After 24 h of incubation with ADQ in 10% FBS media, total cell lysates were harvested and analyzed by immunoblotting. The expression levels of Twist1, E-cadherin, N-cadherin, and α-tubulin were observed by specific antibodies. (**G**) Schematic representation of ADQ inhibiting the Akt pathway and Twist1. Data are representative of three experiments and expressed as the means ± SEM. Data were analyzed by one-way ANOVA followed by Holm-Šídák's post hoc test; *p < 0.05, **p < 0.01, and ***p < 0.001 relative to the ADQ-untreated control.

To further investigate the regulatory mechanisms of ADQ, luciferase reporter assays were performed (Fig. S8). Various promoter activities were measured via the reporter system, including the urinary-type plasminogen activator (uPA), uPA receptor (uPAR), heparanase-1, and E-cadherin promoters. Notably, the E-cadherin promoter activity was significantly increased (Fig. S8D, 5 μM: **p = 0.0032). As the alteration in E-cadherin expression is a hallmark of cancer development, ADQ was further analyzed for its molecular regulatory mechanisms. ADQ was applied for the indicated time, and Twist1 levels were observed along with the level of Akt phosphorylation by immunoblot analysis (Fig. 4A). Twist1 and phospho-Akt levels were decreased by ADQ treatment. The decrease in p-Akt and Twist1 levels was also observed in other liver cancer cell lines, such as Hep3B and Huh7 cells (Fig. S9). In addition, a reduction of the Twist1 expression level was observed earlier than the inhibition of Akt phosphorylation (Fig. S10), which suggests the possibility that Twist1 is regulated by the other pathway or Twist1 itself regulates the phosphorylation of Akt. When the ERK pathway was analyzed with the ADQ treatment, no dramatic change of MAPK pathway was observed by ADQ, whereas sorafenib treatment induced the suppression of ERK phosphorylation as expected (Fig. S11). The overexpression or knockdown of Twist1 showed no effects on either Akt expression or its phosphorylation level (Figs. 4B and S12A). Furthermore, when Akt wild-type or inactive mutant was overexpressed, the endogenous expression levels of Twist1 were not altered (Fig. 4C). These data indicate that the effects of ADQ on Akt phosphorylation and Twist1 expression should be independent.

The suppressive effects of ADQ on Twist1 expression were investigated at the transcriptional and translational levels. The transcriptional levels of *TWIST1* were not altered by ADQ treatment (Fig. 4D). When SK-Hep1 cells were treated with ADQ, the Twist1 protein expression level was down-regulated as observed previously, but its down-regulation was abolished by treatment with MG132, a potent inhibitor of the proteasome (Fig. 4E). Altogether, these data indicate that the decreased Twist1 by ADQ is mediated by the regulation of Twist1 protein degradation rather than its gene expression. When the Twist1 protein level was reduced in the cells treated with ADQ dose-dependently, E-cadherin expression was induced, and N-cadherin expression was suppressed (Fig. 4F). Quantitative analysis of Twist1 target genes also showed significant changes in response to ADQ treatment (Fig. S13A, 5 μM: **p = 0.0032; Fig. S13B, 5 μM: *p = 0.044; Fig. S13C, 5 μM: **p = 0.0076). It was also observed that when the endogenous Twist1 level was decreased with siRNA transfection, cell migration and invasion were inhibited as shown with ADQ treatment (Fig. S12). These data demonstrate that ADQ treatment leads to the inhibition of the Twist1 expression, which results in the alteration of cadherin expressions and the suppression of further progression of cancer (Fig. 4G).

## Discussion

Most cancer mortality can be attributed to metastasis that occurs during cancer progression^20^. Thus, targeting developing cancer provides a novel therapeutic approach. In this study, we identified a potent cancer inhibitor, ADQ, and evaluated its anti-cancer mechanisms at cellular and molecular levels.

The anti-proliferative effects of ADQ were shown in our study to be mediated by suppression of the Akt pathway. Activation of the Akt pathway generally leads to increased proliferation and suppressed apoptosis or autophagy^21^. The FoxO transcription factor is phosphorylated by Akt and then exported from the nucleus^6^. The suppression of Akt phosphorylation by ADQ led to up-regulated FoxO activity, which was observed in luciferase reporter assays and by the expression levels of CDKIs. Increased expression of p21 encoded by the *CDKN1A* gene was observed at both the transcriptional and translational levels. It is a crucial CDKI in FoxO-induced G1 cell cycle arrest^7^. Therefore, the alteration of the cell cycle distribution and subsequent suppression of cell proliferation by ADQ was possibly mediated via the Akt pathway regulation. Although ADQ did not show significant cytotoxicity up to ~ 5 μM, the cell proliferation was inhibited, which showed a notably different pattern that might be affected by the starting cell density of each assay^22^. Interestingly, this cell cycle arrest induced by Akt inhibition/FoxO activation often leads cells to a quiescent state rather than to cell deaths^23,24^. As FoxO has a potential role in suppressing liver fibrosis via cell cycle arrest^25^, ADQ may have effects on preventing hepatic fibrosis.

ADQ rapidly reduced the Twist1 protein level, which raised the possibility that the regulation of Twist1 by ADQ is not dependent on the Akt pathway. The expression of Twist1 did not alter Akt expression and vice versa, suggesting that ADQ regulates the Akt activation and Twist1 stability independently on each other. The protein level of Twist1 was significantly reduced by ADQ treatment in various liver cancer cell lines, including SK-Hep1, Huh7, and Hep3B. Since Twist1 is associated with drug resistance in various cancer types^26–29^, the application of ADQ may have benefits as a novel therapeutic, targeting liver cancer by inhibiting Twist1. Interestingly, a previous study reported an inhibitor that suppressed Twist1 expression level at working concentrations over 10 μM in oncogene-driven lung cancer^30^. Since ADQ showed a sufficient reduction of Twist1 at 5 μM in our study, this suggests that ADQ is efficient in suppressing Twist1 levels and can be developed as a drug candidate for treating multiple cancer types.

ADQ showed potential anti-cancer effects through regulation of the Akt pathway and Twist1 protein level in our study. To further evaluate ADQ in the molecular regulation of the metastasis-associated pathways, the regulatory mechanisms of the Akt pathway and Twist1 by ADQ needs to be verified in different cancer types, which is our next research goal.

## Methods

### Chemical screening

A chemical compound library (~ 3300 compounds) contains (1) compounds extracted from natural products and (2) re-positioning chemical drugs. This library was provided by the Korea Chemical Bank (http://www.chembank.org). The library was screened through wound healing assays of the human liver cancer cell line, SK-Hep1. SK-Hep1 cells (2 × 10^4^ cells/well) were seeded in 96-well plates and incubated overnight. The wound was made by scratching with a pipette tip, and the detached floating cells were removed. Each compound was applied to the cells at 1 μM concentration in 1% FBS media, and the cells were incubated at 37 ℃ for 24 h. The wound area at 0 h and 24 h was observed with a JuLI Stage Real-Time Cell History Recorder. Wound closure was calculated as (X_0_ – X_24_)/X_0_ * 100 (%)_,_ where X_N_ indicates the wound area at N-h point. The values were plotted as a heatmap using “ComplexHeatmap” package in R (version 4.0.1). A literature search was followed using the chemical structures of initially screened compounds provided by the Korea Chemical Bank upon request. ADQ was selected for further evaluation because there were no previous reports of the anti-cancer effects of ADQ.

### Cell culture

SK-Hep1, Huh7, Hep3B, and PLC-PRF-5 cell lines were purchased from the Korean Cell Line Bank (Seoul, Korea). SK-Hep1 and Hep3B cells were grown in Dulbecco’s modified Eagle’s medium (DMEM). Huh7 cells were grown in Roswell Park Memorial Institute (RPMI) 1640 medium, and PLC-PRF-5 cells were grown in RPMI 1640 containing 25 mM HEPES. All cell culture media contains 10% fetal bovine serum (FBS), 50 μg/ml streptomycin, and 50 U/ml penicillin (GIBCO BRL, Grand Island, NY, USA). Cells were grown at 37 ℃, 5% CO_2_.

### Reagents

Rabbit polyclonal anti-Akt (cat. No. sc-8312), rabbit polyclonal anti-p-Akt (Ser473, cat. No. sc-7985), mouse monoclonal anti-Twist1 (cat. No. sc-81417), mouse monoclonal anti-p53 (cat. No. sc-126), rabbit polyclonal anti-p21 (cat. No. sc-397), rabbit polyclonal anti-BAX (cat. No. sc-493), mouse monoclonal FKHRL1/FOXO3a (cat. No. sc-48348), and mouse monoclonal anti-α-tubulin (cat. No. sc-5286) were purchased from Santa Cruz Biotechnology, Inc. (Dallas, TX, USA). Rabbit polyclonal anti-Erk1/2 (cat. No. #9102) and mouse monoclonal anti-p-Erk1/2 (cat. No. #9106) were purchased from Cell Signaling Technology, Inc. (Danvers, MA, USA). Rabbit polyclonal anti-GAPDH (cat. No. GTX100118) was purchased from Gene Tex, Inc. (Irvine, CA, USA). Mouse monoclonal anti-E-cadherin (cat. No. 610182) and mouse monoclonal anti-N-cadherin (cat. No. 610720) antibodies were purchased from BD Biosciences (San Jose, CA, USA). All primary antibodies were diluted at the ratio of 1:1000 in 3% bovine serum albumin. Polyclonal anti-mouse IgG Fc-HRP (cat. No. LF-SA8001) and polyclonal anti-rabbit IgG-HRP (cat. No. LF-SA8002) were purchased from AbFrontier (Young In Frontier Co., Ltd., Seoul, Korea) and diluted at 1:5000. LY294002 (cat. No. #440202-5MGCN, 5 mg) was purchased from Calbiochem (San Diego, CA, USA). Sorafenib (cat. No. SML2653-5MG) was purchased from Sigma–Aldrich (St. Louis, MO, USA).

### Cell viability assay

Cells were seeded in 96-well plates (4 × 10^4^ cells/well) and treated with ADQ for 24 or 48 h with 10% FBS media. Cell viability was evaluated using the EZ‑Cytox solution (Daeil Lab, Seoul, Korea) according to the manufacturer's instructions. The optical densities (OD) of the supernatants were measured at 450 nm using a Synergy H1 Microplate Reader (BioTek Instruments, Winooski, VT, USA).

### Wound healing assay

The cells were seeded in 6-well plates (1.2 × 10^6^ cells/well). The wound was made with a scratcher tip (0.5 mm; SPL Life Sciences, Gyeonggi-do, Korea) 24 h after cell seeding. Then the cells were treated with ADQ in 1% FBS. The wound area was observed at the indicated time point (at the 24 h point or with 6 h interval, up to 36 h) with a JuLI Stage Real-Time Cell History Recorder (NanoEnTek Inc., Seoul, Korea). The wound closure (a distance of the wound) is represented as the percent of wound recovery. All experiments were performed in triplicate.

### Migration and invasion assay

For invasion assay, transwells (24-well type, pore size 8 µm) were coated with Matrigel (0.5 mg/ml; BD Biosciences, Franklin Lakes, NJ, USA) for 2 h. The cells (2 × 10^5^ cells/well) in 1% FBS media were added to the upper chamber along with ADQ, and the lower chamber was filled with 10% FBS media. After incubation at 37 ℃ for 6 h (for migration assay) or 21 h (for invasion assay), the membranes were fixed for 10 min in 3.7% paraformaldehyde. The migrated or invaded cells were stained with 0.5% crystal violet for 10 min. The cells on the upper surface of the membrane were removed with a cotton swab. After being dried, five random fields of the membrane were observed at 100× magnification.

### Gelatin zymography

Cells were seeded in 12-well plates (2 × 10^5^ cells/well) for 16 h. ADQ was added to the cells in serum-free media for 24 h. The collected media were mixed with 5× sample buffer without β-mercaptoethanol. The samples were analyzed by gelatin zymography using 0.1% gelatin-containing 10% acrylamide SDS-PAGE. After gel electrophoresis, the gel was washed with the wash buffer [2.5% Triton X-100, 50 mM Tris–HCl (pH 7.5), 5 mM CaCl_2_, 1 μM ZnCl, 0.016% NaN_3_] for 30 min at room temperature (RT). Then, the gel was incubated in the substrate buffer [1% Triton X-100, 50 mM Tris–HCl (pH 7.5), 5 mM CaCl_2_, 1 μM ZnCl, 0.016% NaN_3_] for 24 h at 37 °C. For the visualization, the gel was stained with Coomassie Blue for 30 min at RT and then destained with a methanol/acetic acid solution for 1 h at RT.

### MMP activity assay

SK-Hep1 cells were seeded in 12-well culture plates. The cultured medium changed to serum-free medium containing ADQ with indicated concentration. The medium was collected after 24 h. The collected medium was incubated with an assay system according to the manufacturer’s instructions of EnzChek Gelatinase/Collagenase Assay Kit (E12055, Thermo Fisher Scientific, Inc., USA). The fluorescence was measured with (microplate reader).

### Soft-agar colony formation assay

SK-Hep1 cells (4500 cells/well) were mixed with 0.3% agarose (Sigma–Aldrich), and the mixture was seeded on 0.5% agarose in 12-well plates. The media with ADQ was replaced every 3 days. After incubation for 14 days, the colonies were stained with a 0.05% crystal violet solution. The stained samples were photographed by a digital camera (Olympus SP-350; Cam2Com).

### MTS invasion assay

SK-Hep1 cells were seeded in low-attachment 96-well plates (2000 cells/well) and incubated for 3 days. After media removal, Matrigel (3 mg/ml; BD Biosciences, Franklin Lakes, NJ, USA) was added. The plate was centrifuged at 300*g* for 3 min at 4 °C and then incubated at 37 ℃ and 5% CO_2_ overnight. The spheroids were treated with ADQ in 10% FBS media. All images were captured by a JuLI Stage Real-Time Cell History Recorder (NanoEnTek Inc., Seoul, Korea). The cross-sectional areas of spheroids were measured using ImageJ (https://imagej.nih.gov/ij/). The invading area was calculated as a ratio to the size of the spheroid at the 0 h time point; (Area_N_ – Area_0_)/Area_0_.

### Immunoblot analysis

After the cells were treated with ADQ for 24 h or transfected with the indicated plasmid for 48 h, cell lysates were harvested with lysis buffer [20 mM Tris‑HCl (pH 8.0), 150 mM NaCl, 0.5% Triton X‑100, 0.5% IGEPAL CA‑630, 1 mM EDTA, 1% glycerol, 1 mM phenylmethylsulfonyl fluoride, 10 mM NaF, and 1 mM Na_3_VO_4_]. The protein concentrations were determined by a Bradford protein assay (Bio‑Rad Laboratories, Inc.) according to the manufacturer's protocol. Equal amounts of protein samples were boiled in 5× SDS sample buffer [12 mM Tris–HCl (pH 6.8), 5% glycerol, 0.4% SDS, 1% β-mercaptoethanol, and 0.02% bromophenol blue] at 100 °C for 5 min. Samples were separated by 12% SDS‑PAGE and transferred onto nitrocellulose membranes. The membranes were blocked with 5% nonfat‑dried skimmed milk for 1 h at 25 °C and then they were incubated for 16 h at 4 °C with a specific primary antibody diluted in 3.3% BSA in 1 × TBST [Tris‑buffered saline with 0.05% Tween 20]. The membranes were washed several times with 1× TBST and then incubated with the appropriate secondary antibody for 2 h at 25 °C. Protein bands were detected by enhanced chemiluminescence immunoblotting detection reagent (Pierce; Thermo Fisher Scientific, Inc.). All immunoblot analysis was performed at least three times. The band intensities of each protein were analyzed with ImageJ (https://imagej.nih.gov/ij/).

### Reverse transcription-polymerase chain reactions (RT-PCR) analysis

SK-Hep1 cells were seeded in 12-well plates (2 × 10^5^ cells/well). Cells were treated with ADQ (0, 2, and 5 μM) for 24 h. Total RNA was prepared using the Accuzol total RNA extraction solution (Bioneer Corporation, Daejeon, Korea). Total RNA (1 μg) was reverse transcribed into complementary DNA (cDNA) using a TOPscript cDNA synthesis kit (ENzynomics, Daejeon, Korea). Briefly, the amplification conditions were as follows: 95 °C (5 min) initial denaturation followed by 25 cycles of 95 °C for 5 s and annealing/extension at 55 °C (30 s). The gene expression levels were normalized relative to the reference gene, glyceraldehyde 3-phosphate dehydrogenase (*GAPDH*). Primer sequences used are described here. *GAPDH* sense, 5ʹ-gtcttcaccaccatggagaagg-3ʹ; antisense, 5ʹ-cctgcttcaccaccttcttgat-3ʹ; *CDKN1A* sense, 5ʹ- cagtggacagcgagcagctgag-3ʹ; antisense, 5ʹ-ggacccttcagcctgctccc-3ʹ; *CDKN1B* sense, 5ʹ-taattggggctccggctaact-3ʹ; antisense, 5ʹ-ccctcccttccccaaagttta-3ʹ; *CDKN2B* sense, 5ʹ-cgtgggaaagaagggaagagt-3ʹ; antisense, 5ʹ-aacggttgactccgttgggat-3ʹ ; *CDKN2D* sense, 5ʹ-ctgatgtcaacgtgcctgatg-3ʹ; antisense, 5ʹ-gggcaggagaaacaagaagag-3ʹ; *TWIST1* sense, 5ʹ-acgtgtccagctcgccagtc-3ʹ; antisense, 5ʹ-tggagtccagctcgtcgctc-3ʹ.

### qRT-PCR analysis

Total SK-hep1 cells RNA and cDNA was prepared following RT-PCR method. SYBR Green Q Master mix (COSMO Genetech, Seoul, Korea) were used to perform qRT-PCR with CFXConnect real-time thermal cycler (Bio-Rad, Hercules, CA, USA). cDNA was initially denatured at 95 °C (5 min) and then amplified by following 39 cycles of at 95 °C for 5 s and 60 °C for 30 s. The PCR amplicon fluorescence value were normalized relative to *GAPDH* and expressed as the ratio of gene expression levels by referring to the untreated group as 100%. It was according to the 2−∆∆Cq method. Primer sequences used are described here. *GAPDH* sense, 5ʹ-ggtgtgaaccatgagaagtatga-3ʹ; antisense, 5ʹ-gagtccttccacgataccaaag-3ʹ; *CDKN1A* sense, 5ʹ-aggtggacctggagactctcag-3ʹ; antisense, 5ʹ-tcctcttggagaagatcagccg-3ʹ; *CDKN1B* sense, 5ʹ-ataaggaagcgacctgcaaccg-3ʹ; antisense, 5ʹ-ttcttgggcgtctgctccacag-3ʹ; *CDKN2B* sense, 5ʹ-acggagtcaaccgtttcgggag-3ʹ; antisense, 5ʹ-ggtcgggtgagagtggcagg-3ʹ ; *CDKN2D* sense, 5ʹ-gtgcatcccgacgccctcaac-3ʹ; antisense, 5ʹ-tggcaccttgcttcagcagctc-3ʹ; *CDH1* sense, 5ʹ-tacactgcccaggagccaga-3ʹ; antisense, 5ʹ-tggcaccagtgtccggatta-3ʹ; *CDH2* sense, 5ʹ-cctccagagtttactgccatgac-3ʹ; antisense, 5ʹ-gtaggatctccgccactgattc-3ʹ; *VIM* sense, 5ʹ-aggcaaagcaggagtccactga-3ʹ; antisense, 5ʹ-atctggcgttccagggactcat-3ʹ.

### Cell cycle analysis

SK-Hep1 cells (1 × 10^6^ cells) were treated with ADQ for 24 h in 10% FBS media. Harvested cells were fixed in 70% cold ethanol at − 20 °C for 3 h. The fixed cells were washed with PBS and stained with 50 μg/ml of propidium iodide (Sigma–Aldrich, St. Louis, MO, USA) for 30 min at 25 °C in the dark. The DNA content of each sample was analyzed by a Guava Muse Cell Analyzer (Luminex Corporation, Austin, TX, USA).

### Cell growth assay

SK-Hep1 cells (1500 cells/well) were seeded into 96-well plates. ADQ (0, 2, and 5 µM) was added in 10% FBS media for 96 h. Cell growth was evaluated using the EZ‑Cytox solution according to the manufacturer's instructions.

### Apoptosis assay

SK-Hep1 cells (1 × 10^6^ cells) were seeded in 6-well plate. The cells were treated with ADQ for 24 h in 10% FBS media. Harvested cells were washed twice with ice-cold PBS. The rate of apoptotic cells was evaluated using FITC Annexin V (BioLegend, San Diego, CA, USA). Annexin V-FITC/PI double staining method was performed according to the manufacturer’s instruction. Stained cells were analyzed by FACSCalibur flow cytometer (BD Biosciences, San Jose, CA, USA). Acquired data from the flow cytometer were analyzed using the CellQuest analysis software (BD Biosciences, San Jose, CA, USA).

### p21 siRNA transfection

p21 siRNA and scrambled negative control were purchased from Bioneer (Daejeon, Korea). p21 siRNA sense, 5ʹ-CUG UAC UGU UCU GUG UCU U-3ʹ; antisense, 5ʹ-AAG ACA CAG AAC AGU ACA G -3ʹ; scrambled negative control sense, 5ʹ-CCU ACG CCA CCA AUU UCG U-3ʹ; antisense, 5ʹ-ACG AAA UUG GUG GCG UAG G-3ʹ. A mixture of 100 nM siRNA and 5 μl Lipofectamine 2000 (Thermo Fisher Scientific Inc, MA, USA) was incubated in serum-free media for 30 min at RT. After incubation, the mixture was added to SK-Hep1 cells for siRNA transfection.

### Subcellular fractionation

SK-Hep1 cells were treated with ADQ for 24 h. Before fractionation, cells were washed and collected with ice-cold PBS into 1.5 ml microcentrifuge tubes. The cells were harvested by centrifugation at 1300 rpm for 5 min at 4 °C. After removing the supernatant, ice-cold 0.1% NP40 (Calbiochem, San Diego, CA, USA) was added and the pellet was suspended. The samples were centrifuged at 8000 rpm for 1 min at 4 °C. After centrifugation, nuclei were sedimented and the supernatant containing the cytoplasmic fraction was transferred into new 1.5 ml microcentrifuge tubes. For cytosolic fractionation, 5× SDS was then added to the supernatants and boiled at 100 °C for 5 min. For the collection of nucleic fractions, the pellet was resuspended in ice-cold NP40 and centrifuged briefly for 1 min at 4 °C. 1× SDS was added to the nucleic fractions after removing the supernatant. Nucleic fractions were then sonicated twice using microprobes at 30% level for 3 s and boiled at 100 °C for 10 min.

### MTS growth assay

SK-Hep1 cells (2000 cells/well) were seeded on 1% agarose-coated 96-well plates. The cells were incubated for 16 h, and then the formed spheroids were treated with ADQ in 10% FBS media for 96 h. Images were captured with 24 h interval by a JuLI Stage Real-Time Cell History Recorder. The cross-sectional area of spheroids at each time point was measured using ImageJ. The spheroid growth was plotted as a ratio (%) of spheroid size at each time point to the spheroid size at the 0 h point.

### Luciferase reporter assay

SK-Hep1 cells (5 × 10^6^ cells/well) were seeded in 100-mm dishes and co-transfected with a pE-cadherin-Luc cis-reporter plasmid (Agilent Technology Inc., Santa Clara, CA, USA) and a gWIZ-GFP plasmid. The other luciferase reporter plasmids containing the uPAR (− 682/+ 27) (pcDNA3.1-uPAR (− 682/+ 27)-Luc), uPA (− 810/+ 30) (pcDNA3.1-uPA (− 810/+ 30)-Luc), or HPR1 (− 462/+ 98) promoter region (pcDNA3.1-HPR1(− 462/+ 98)-Luc) were constructed with the insertion of PCR products into the BglII and EcoRI sites of the plasmid pcDNA3.1-Luc. The pNF-κB‑Luc or pAP1‑Luc reporter plasmids (Agilent Technologies Inc.) contained the NF-κB or AP1 promoter, respectively. In addition, the pSOD-luc plasmid was a kind gift from Prof. Jongkyeong Chung (Seoul National University, Seoul, Korea). Transfected cells were divided into 12-well plates (1 × 10^5^ cells/well) and incubated for 24 h. Then the cells were treated with the indicated concentrations of the compound for an additional 24 h. The cells were lysed with Cell culture lysis reagent (Promega Corporation, Fitchburg, WI, USA), and luciferase activities were measured according to the instructions of the manufacturer (Promega, Madison, WI, USA). The luciferase activities were normalized against the level of GFP expression.

### Twist1 siRNA transfection

Twist1 siRNA and scrambled negative control were purchased from Bioneer (Daejeon, Korea). Twist1 siRNA #1 sense, 5ʹ-CUG AAC AGU UGU UUG UGU U-3ʹ; antisense, 5ʹ-AAC ACA AAC AAC UGU UCA G-3ʹ; #2 sense, 5ʹ-GGA CCC AUG GUA AAA UGC A; antisense, 5ʹ-UGC AUU UUA CCA UGG GUC C; #3 sense, 5ʹ-GCG CUU UCU UUU UGG ACC U; antisense, 5ʹ-AGG UCC AAA AAG AAA GCG C; scrambled negative control sense, 5ʹ-CCU ACG CCA CCA AUU UCG U-3ʹ; antisense, 5ʹ-ACG AAA UUG GUG GCG UAG G-3ʹ. A mixture of 100 nM siRNA and 5 μl Lipofectamine 2000 (Thermo Fisher Scientific Inc, MA, USA) was incubated in serum-free media for 30 min at RT. After incubation, the mixture was added to SK-Hep1 cells for siRNA transfection.

### Statistical analysis

All data are expressed as means ± standard error of the mean (SEM). Statistical analysis of the data was performed by one-way ANOVA using Prism 9 (GraphPad Software, San Diego, CA, USA) and post-hoc analysis was performed using Holm-Šídák's method. *p < 0.05, **p < 0.01, and ***p < 0.001 were considered to be statistically significant.

## Supplementary Information

Supplementary Information.

## Data Availability

All data generated or analyzed during this study are included in this published article (and its Supplementary Information files).
