# Supplementary material for: Identification of a novel inhibitor of liver cancer cell invasion and proliferation through regulation of Akt and Twist1
Source: Sci Rep. 2021 Aug 18;11:16765. doi: 10.1038/s41598-021-95933-4 (PMC8373934; doi:10.1038/s41598-021-95933-4)

Supplementary information for

Identification of a novel inhibitor of liver cancer cell invasion and proliferation through regulation of Akt and Twist1

Jain Ha[1, 4], Sewoong Lee [1, 4], Jiyoung Park[1], Jihye Seo[1], Eunjeong Kang[1], Haelim Yoon[1], Ba Reum Kim[1], Hyeon Kyu Lee[2], Seong Eon Ryu[3, *] and Sayeon Cho[1, *]

[1]Laboratory of Molecular and Pharmacological Cell Biology, College of Pharmacy, Chung-Ang University, Seoul 06974, Republic of Korea

[2]Korea Chemical Bank, Korea Research Institute of Chemical Technology, P.O. Box 107, Yuseong, Daejeon 34114, Republic of Korea

[3]Department of Bioengineering, College of Engineering, Hanyang University, Seoul 04763, Republic of Korea

[4]These authors contributed equally: Jain Ha and Sewoong Lee

* **Corresponding Author's Information:** Sayeon Cho (Ph.D.); sycho@cau.ac.kr; Tel.: +82-2-820-5595 or Seong Eon Ryu (Ph.D.); ryuse@hanyang.ac.kr; Tel: +82-2-2220-4022

Supplementary figures

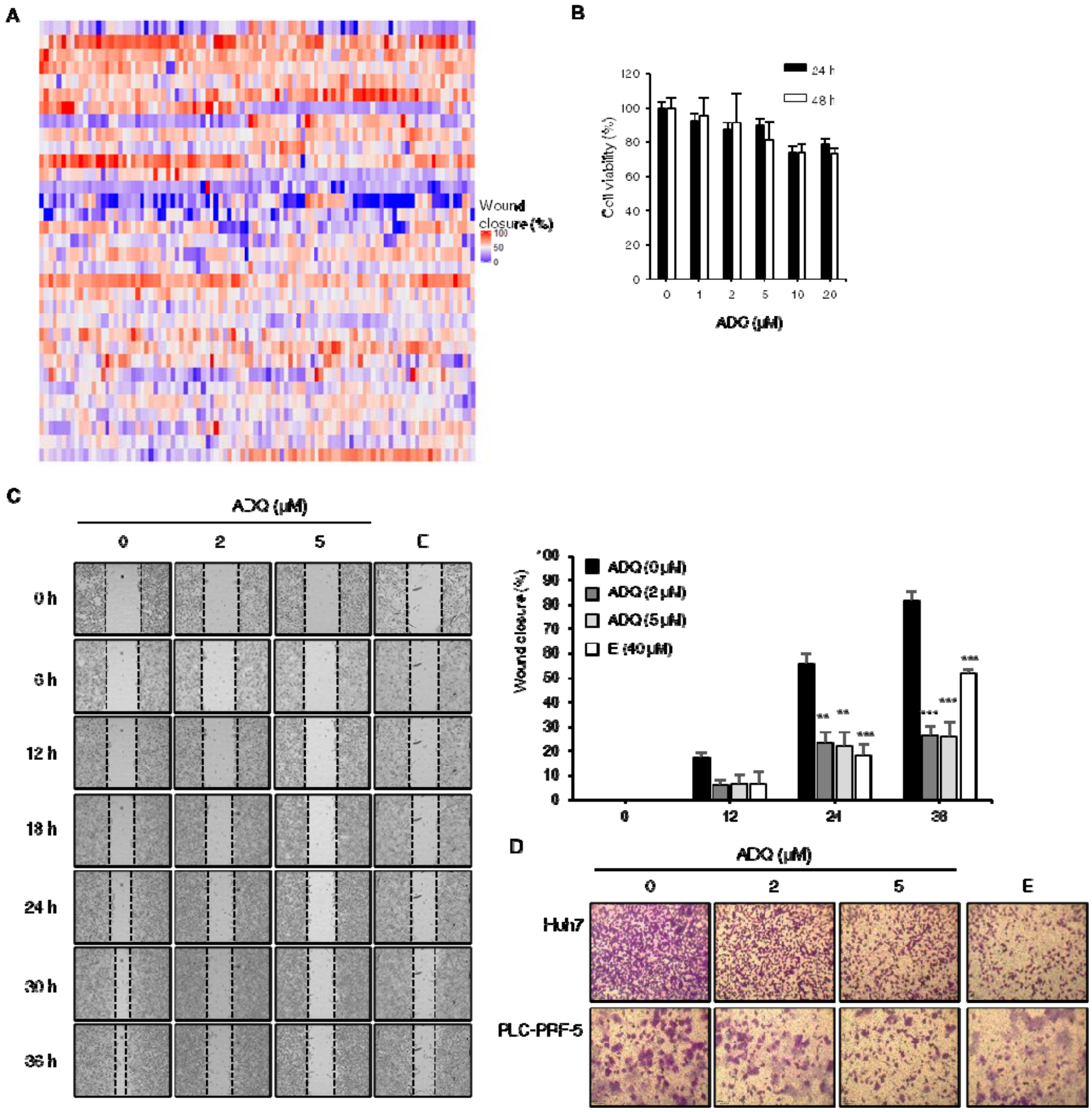

Figure S1. ADQ was screened as a potential anti-cancer inhibitor. (A) Wound closure heatmap of 3,300 compounds. (B) SK-Hep1 cells were treated with ADQ at the indicated concentrations for 24 or 48 h in a cell culture medium containing 10% FBS. The relative cell viability is shown as bar graphs compared to the ADQ-untreated group (100%). Data are

representative of three experiments and expressed as the means ± SEM and analyzed by one-way ANOVA followed by Holm-Šídák's post hoc test; $^*p < .05$, $^{**}p < .01$, *and* $^{***}p < .001$ relative to the ADQ-untreated control. (C) Cell images were taken every 6 h. The wound closure values were quantified by measuring the percent of wound size at each time point, and the relative wound closure (relative to the starting wound size at 0 h) is shown as a bar graph. Data are representative of three experiments and expressed as the means ± SEM and analyzed by one-way ANOVA followed by Holm-Šídák's post hoc test; $^*p < .05$, $^{**}p < .01$, *and* $^{***}p < .001$ relative to the ADQ-untreated control. (D) Huh7 or PLC-PRF-5 cells were treated with ADQ or emodin (E; 20 μM; positive control), and then the invasion assay was performed. After incubation for 21 h, cells invading the lower surface of the chambers were fixed, stained, and imaged.

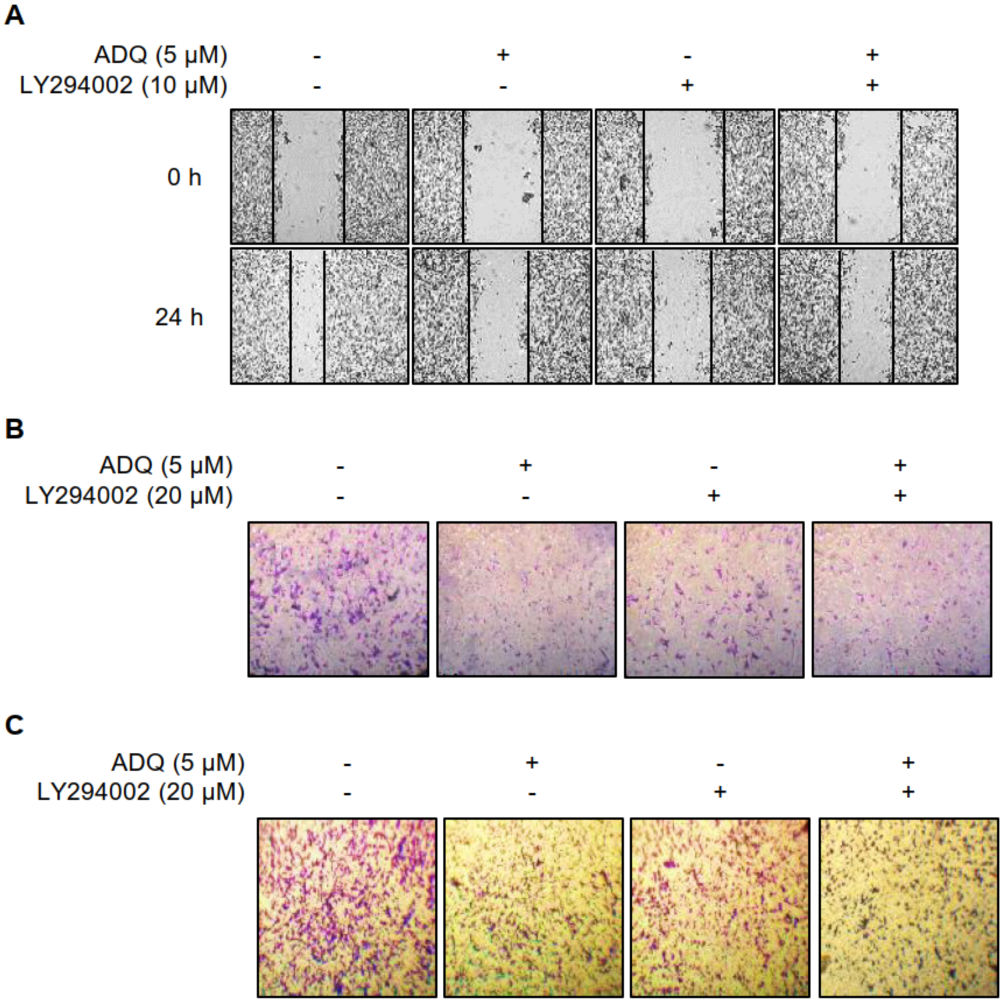

Figure S2. Treatment with an Akt inhibitor results in suppressed cancer physiology similar to ADQ treatment. SK-Hep1 cells were treated either with ADQ or LY294002, an inhibitor of the PI3K/Akt pathway. (A) Wound healing assay, (B) migration assay, and (C) invasion assay were performed as described in Methods. Data are representative of three experiments.

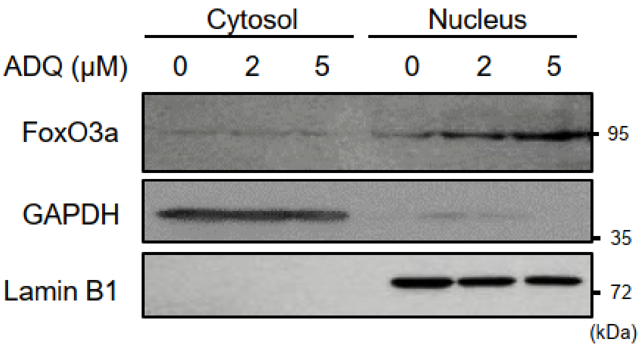

Figure S3. FoxO3, one of Akt downstream substrates localized in the nucleus after ADQ treatment. SK-Hep1 cells were treated with different concentrations of ADQ (0, 2, and 5 μM) in 10% FBS-containing media. Cells were lysed and fractionated into cytoplasmic and nuclear fractions. Each fraction was immunoblotted using specific antibodies. Anti-GAPDH and anti-Lamin B1 antibodies were used to confirm the cytoplasmic and nuclear fraction, respectively.

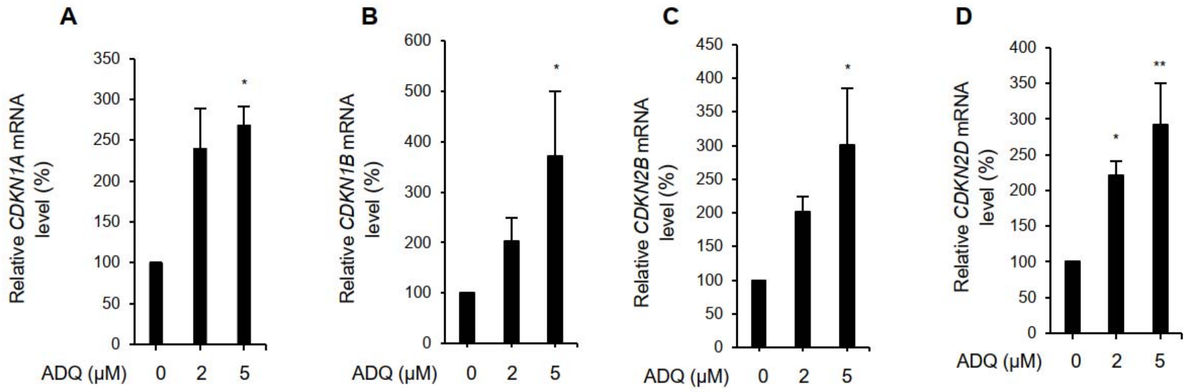

Figure S4. The expression levels of target genes of the Akt/FoxO pathway were analyzed by qRT-PCR. The relative ratio of mRNA levels of (A) *CDKN1A*, (B) *CDKN1B*, (C) *CDKN2B*, and (D) *CDKN2D* were normalized to *GAPDH*. Data are expressed as the means ± SEM (n=3). Data were analyzed by one-way ANOVA followed by Holm-Šídák's post hoc test; *$*p < .05$, $**p < .01$, and $***p < .001$* relative to the ADQ-untreated control.

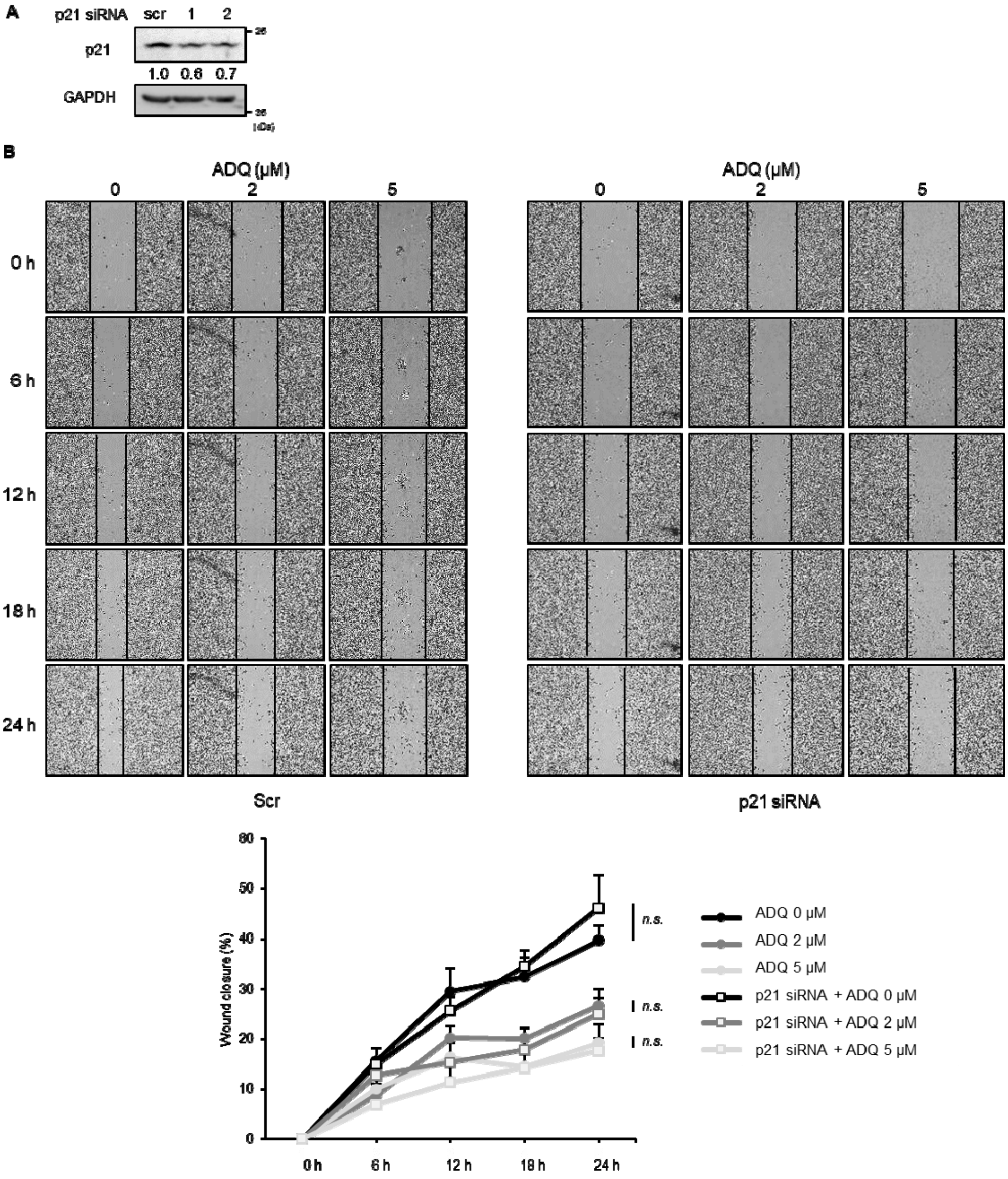

Figure S5. The suppression of p21 expression did not alter the anti-migratory effects of the ADQ treatment. SK-Hep1 cells were transfected with scrambled siRNA ("Scr") or p21 siRNA (100 nM) with Lipofectamine for 48 h. (A) The expression levels of p21 and GAPDH were detected by specific antibodies. (B) The wound was created using SPL Scratcher, and then the

cells transfected with scrambled siRNA or p21 siRNA-1 were incubated with ADQ (0, 2, and 5 μM) in 1% FBS media. Microscopic images were captured for every 6 h. The wound closure values were quantified by measuring the percent of wound size compared to the 0 h point of each sample by ImageJ software. Data are representative of three experiments and expressed as the means ± SEM. Data were analyzed by one-way ANOVA followed by Holm-Šídák's post hoc test; $^{*}p < .05$, $^{**}p < .01$, *and* $^{***}p < .001$ relative to the "scrambled" groups. *n.s.*, not significant.

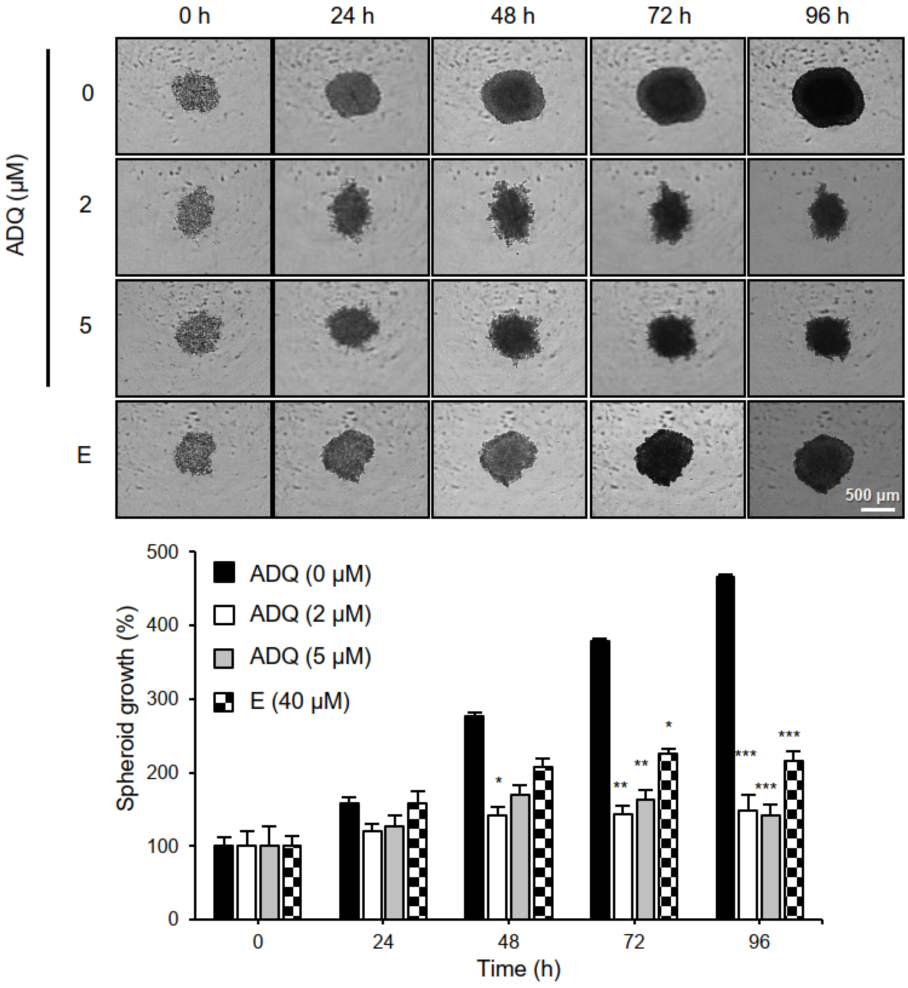

Figure S6. Cell proliferation was inhibited by ADQ in the 3D system. SK-Hep1 cells were seeded in a round bottom low attachment 96-well plate and were incubated with ADQ (0, 2, and 5 μM) or emodin (E; 40 μM; positive control) for 96 h in 10% FBS media. Microscopic images were captured by a JuLI stage real-time imaging system at the indicated time points. The scale bar is 500 μm. Data are representative of three experiments and expressed as the means ± SEM. Data were analyzed by one-way ANOVA assay followed by Holm-Šídák's post hoc test; **p < .05, **p < .01, and ***p < .001* relative to the ADQ-untreated control at each time point.

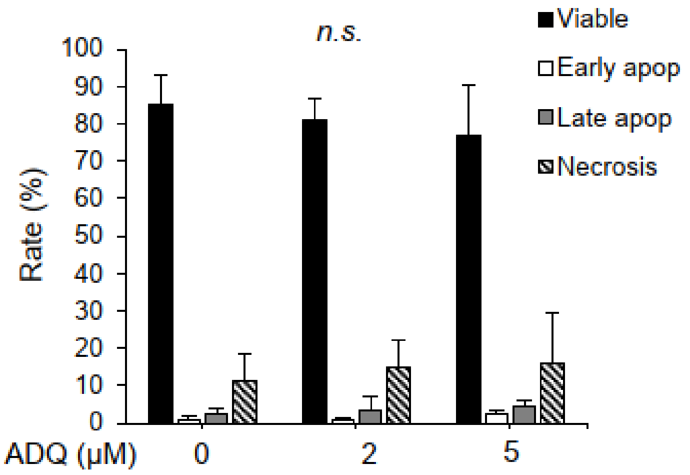

Figure S7. ADQ treatment did not induce apoptosis in SK-Hep1 cells. SK-Hep1 cells were treated with ADQ (0, 2, and 5 μM) in 10% FBS media and analyzed. A bar graph was used to indicate the populations of viable, apoptotic, and necrotic cells among the total cells. Data are expressed as the means ± SEM (n=4). Data were analyzed by one-way ANOVA followed by Holm-Šídák's post hoc test; $*p < .05$, $**p < .01$, *and* $***p < .001$ relative to the ADQ-untreated control. *n.s.*, not significant

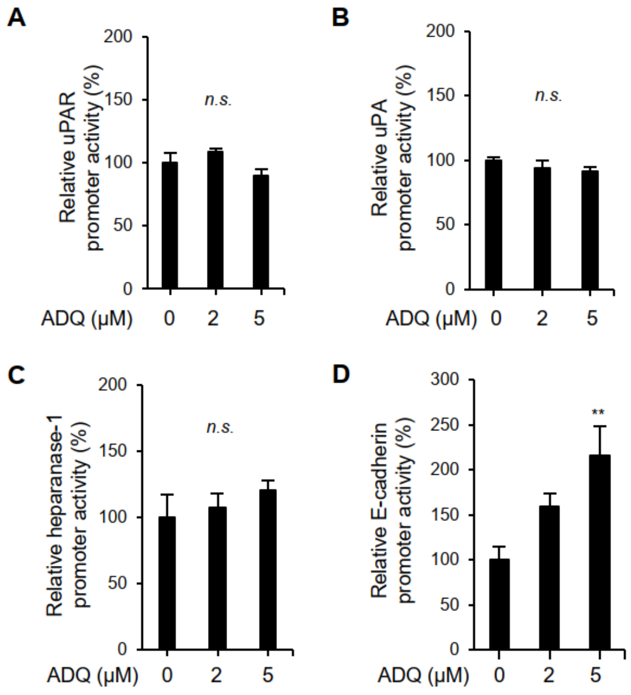

Figure S8. E-cadherin promoter activity was upregulated by ADQ. SK-Hep1 cells were co-transfected with (A) uPAR-, (B) uPA-, (C) heparanase-1-, or (D) E-cadherin-Luc reporter plasmid, and gWIZ-GFP. The cells were treated with the indicated concentrations of ADQ for 24 h in culture media containing 10% FBS. The measured luciferase activities were normalized against the GFP levels. Data are representative of three experiments and expressed as the means ± SEM. Data were analyzed by one-way ANOVA assay followed by Holm-Šídák's post hoc test; **p < .05, **p < .01, and ***p < .001* relative to the ADQ-untreated control.

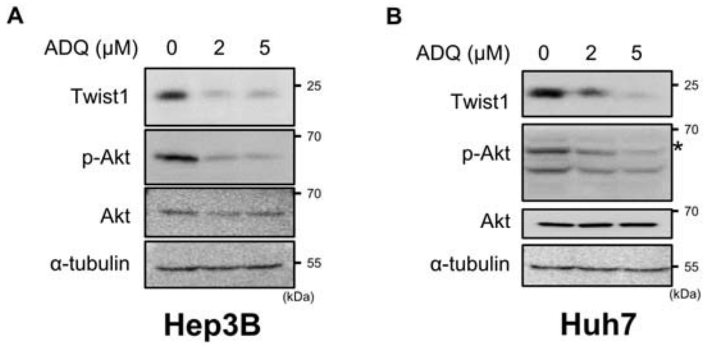

Figure S9. ADQ suppressed Twist1 and Akt phosphorylation in other liver cancer cell lines. (A) Hep3B and (B) Huh7 cells were treated with the indicated concentrations of ADQ for 24 h. The asterisk indicates a p-Akt band. The expression levels of Twist1, p-Akt (Ser473), Akt, and α-tubulin were detected by specific antibodies. Data are representative of three experiments.

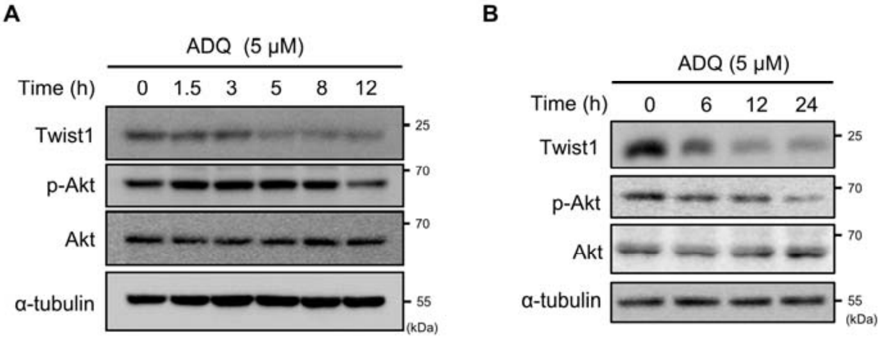

Figure S10. The suppression of Twist1 protein levels was observed earlier than the inhibition of Akt phosphorylation by ADQ treatment. (A) SK-Hep1 or (B) Hep3B cells were treated with ADQ (5 μM) in 10% FBS-containing media and were harvested at the indicated time points. The expression levels of Twist1, p-Akt (Ser473), Akt, and α-tubulin were detected by specific antibodies.

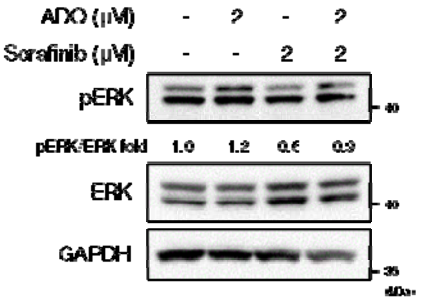

Figure S11. ADQ and sorafenib showed different effects on ERK phosphorylation. ADQ and sorafenib were treated at the indicated concentration to SK-Hep1 cells for 24 h. The protein levels of pERK (pThr202/Tyr204), ERK, and GAPDH were detected by specific antibodies.

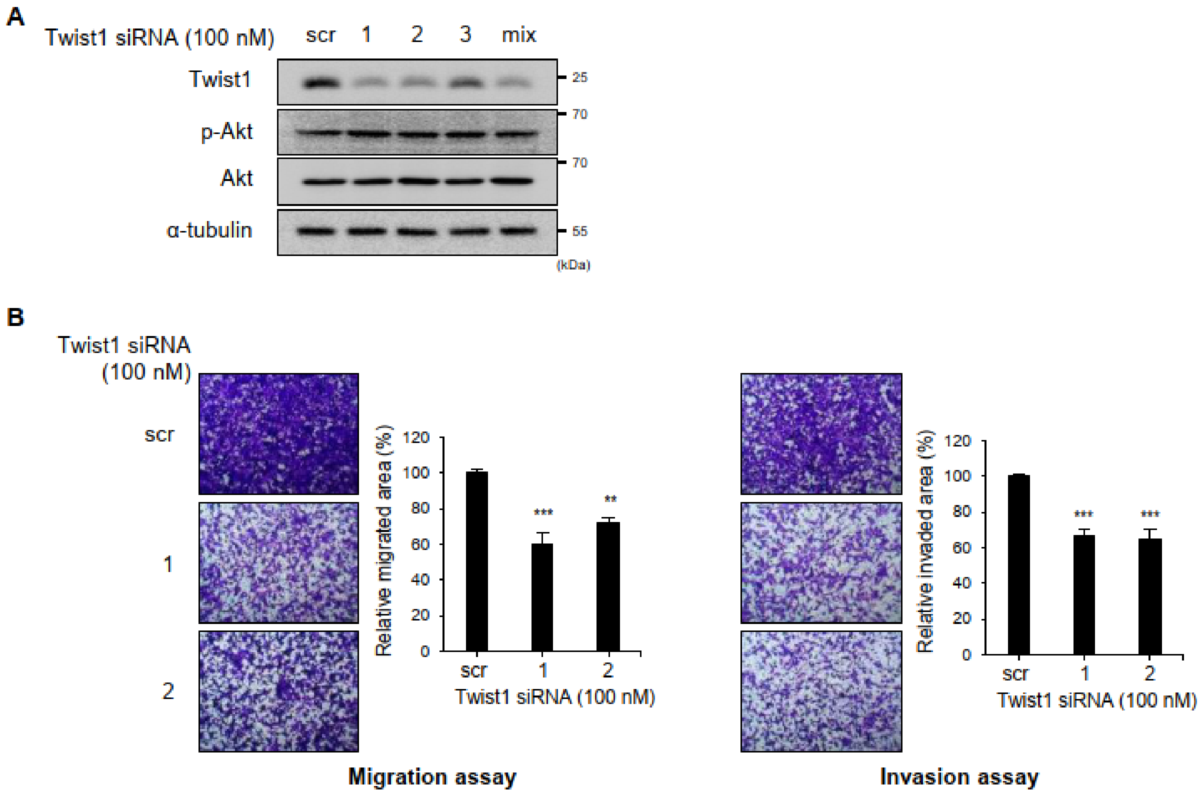

Figure S12. The suppressed invasion of SK-Hep1 cells by ADQ was abolished by Twist1 knockdown. (A) SK-Hep1 cells were transfected with Twist1 siRNA (100 nM) with Lipofectamine for 24 h. The expression levels of Twist1, p-Akt (Ser473), Akt, and α-tubulin were detected by specific antibodies. Data are representative of three experiments. scr, scrambled siRNA (control for siRNA transfection); mix, the mixture of Twist1 siRNA-1, 2, and 3 at the final concentration of 100 nM. (B) The migration or invasion assay was performed in SK-Hep1 cells either transfected with scrambled control siRNA or Twist1 siRNA-1 (100 nM). Data are representative of three experiments and expressed as the means ± SEM. Data were analyzed by one-way ANOVA followed by Holm-Šídák's post hoc test; *$*p < .05$, $**p < .01$, and $***p < .001$* relative to the scrambled-transfected control. Scr, scrambled siRNA (control for siRNA transfection).

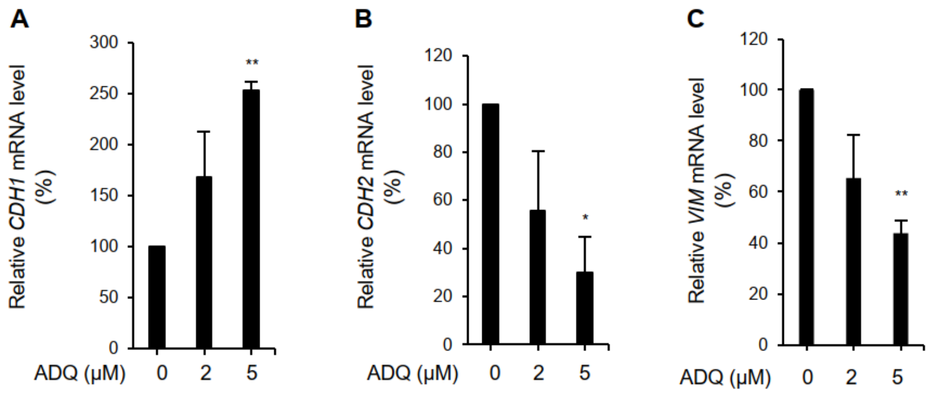

Figure S13. The expression levels of Twist1 target genes were analyzed by qRT-PCR. The relative ratio of mRNA levels of (A) *CDH1*, (B) *CDH2*, and (C) *VIM* were normalized to *GAPDH*. Data are expressed as the means ± SEM (n=3). Data were analyzed by one-way ANOVA followed by Holm-Šídák's post hoc test; $*p < .05$, $**p < .01$, and $***p < .001$ relative to the ADQ-untreated control.

Original images of gels(kb)/blots(kDa)

Figure 1D

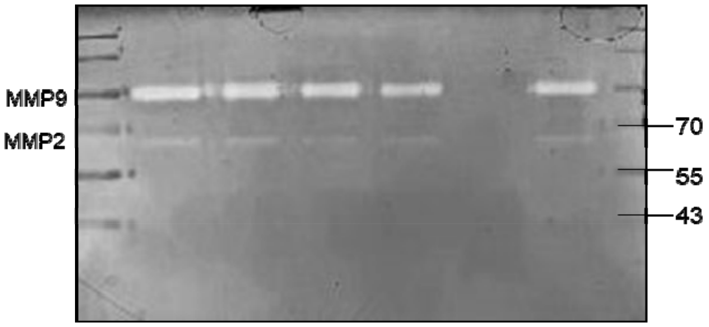

Figure 3A

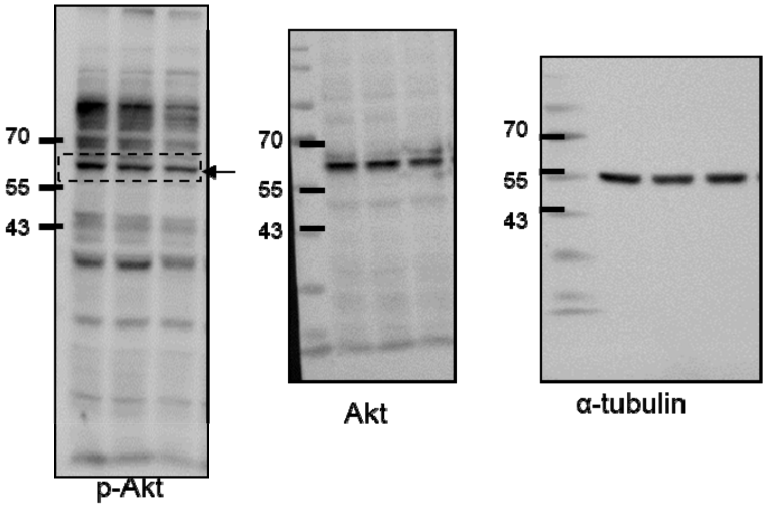

Figure 3B

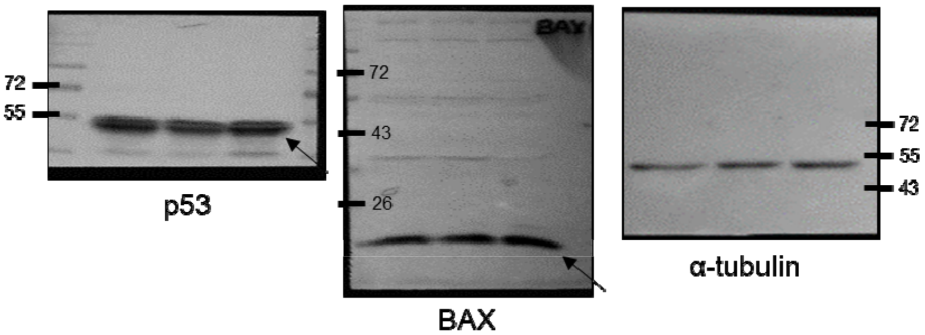

Figure 3D

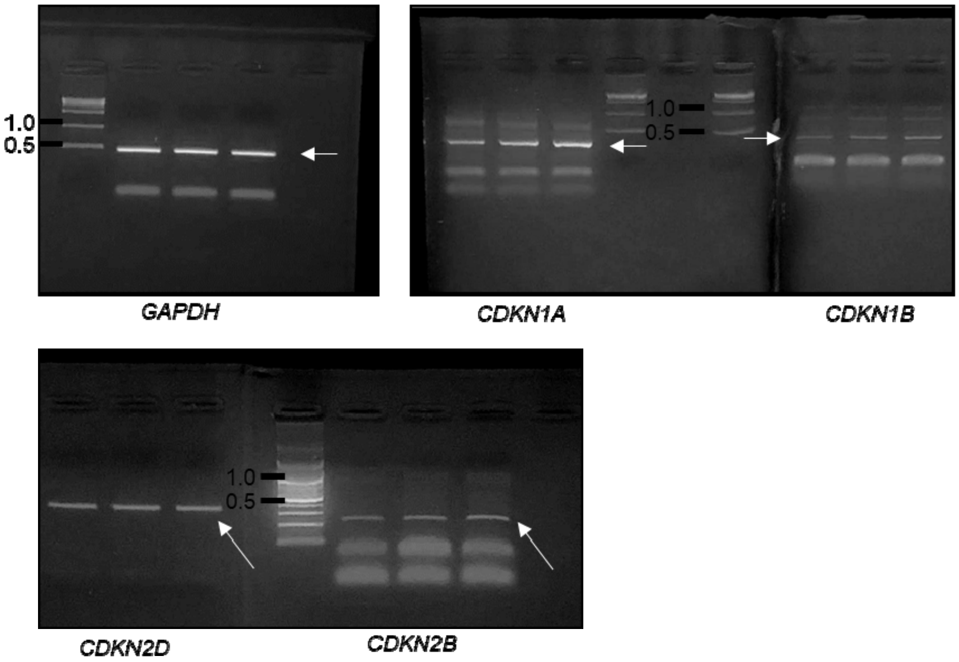

Figure 3E

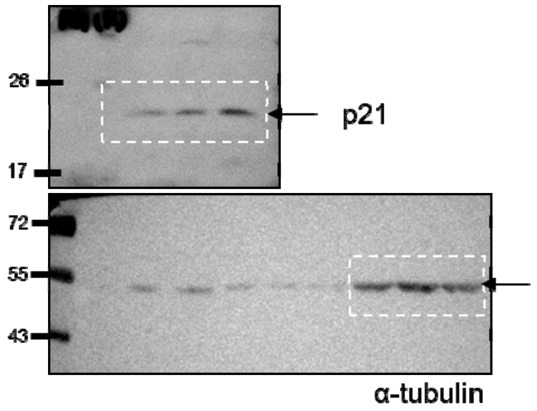

Figure 4A

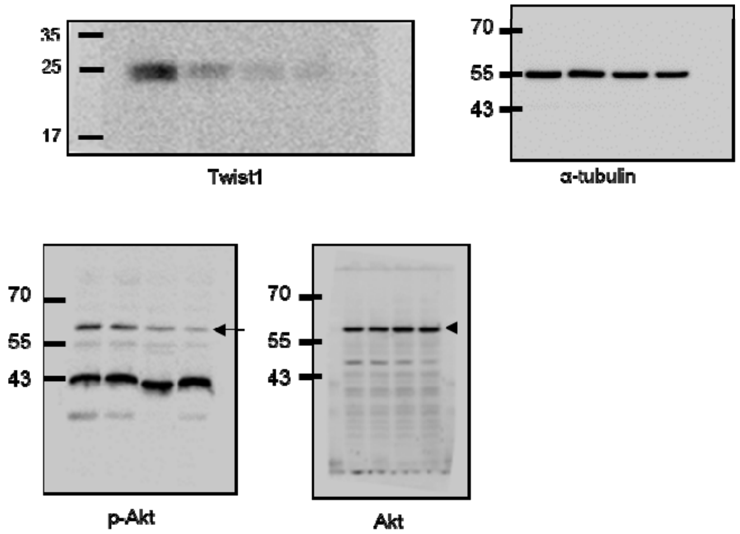

Figure 4B

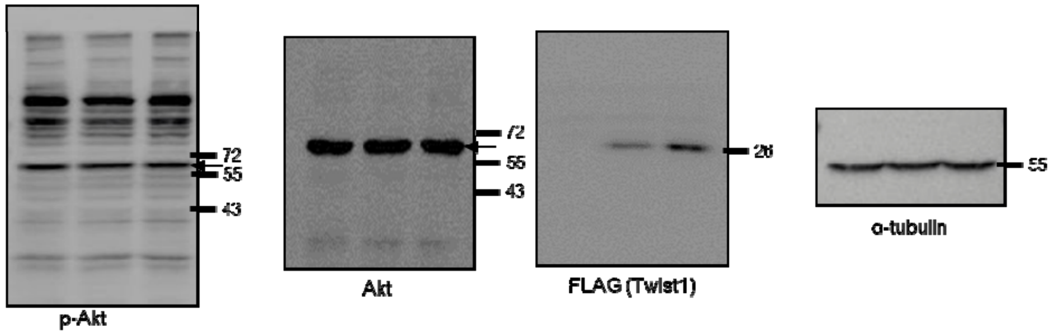

Figure 4C

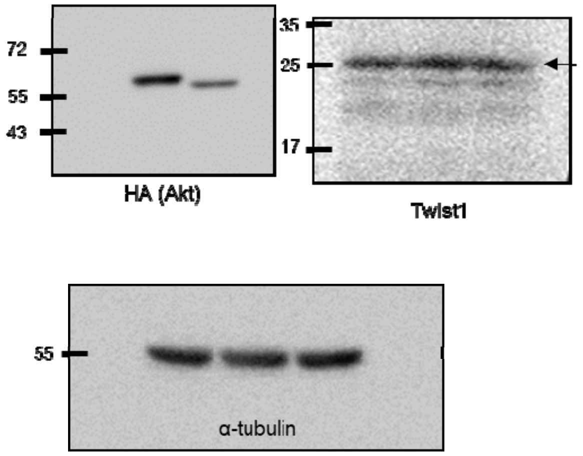

Figure 4D

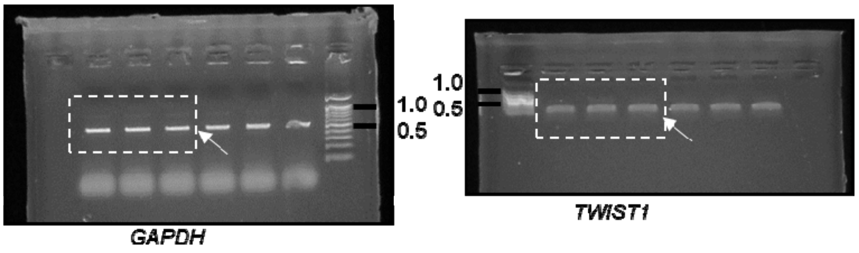

Figure 4E

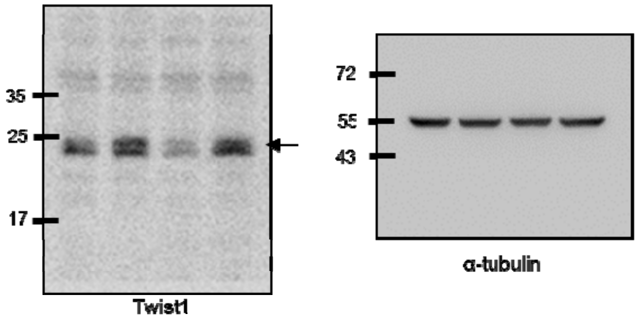

Figure 4F

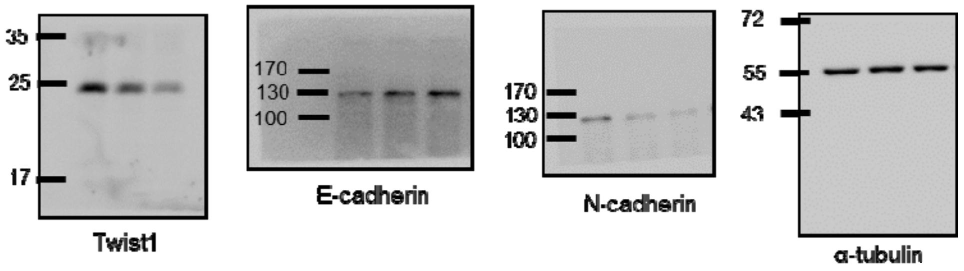
35
25
17
Twist1
170
130
100
E-cadherin
170
130
100
N-cadherin
72
55
43
α-tubulin

Supplementary Figure S3

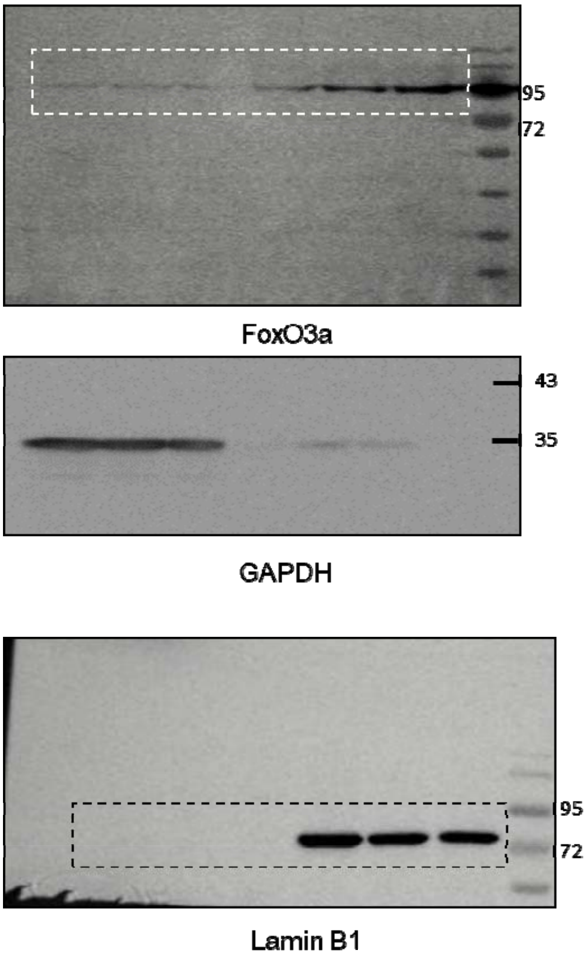

Supplementary Figure S5A

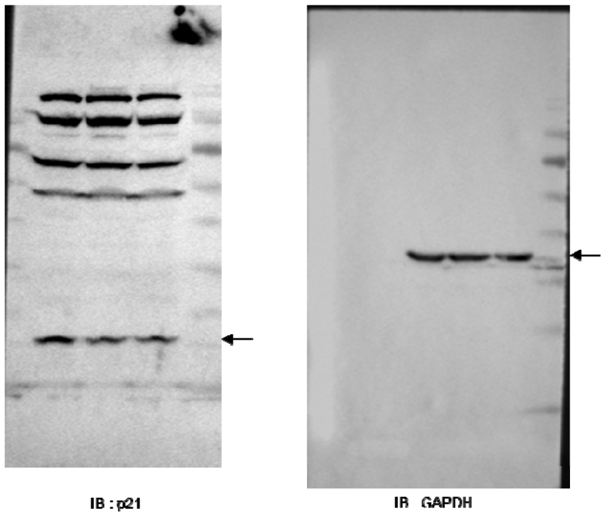

Supplementary Figure S9A

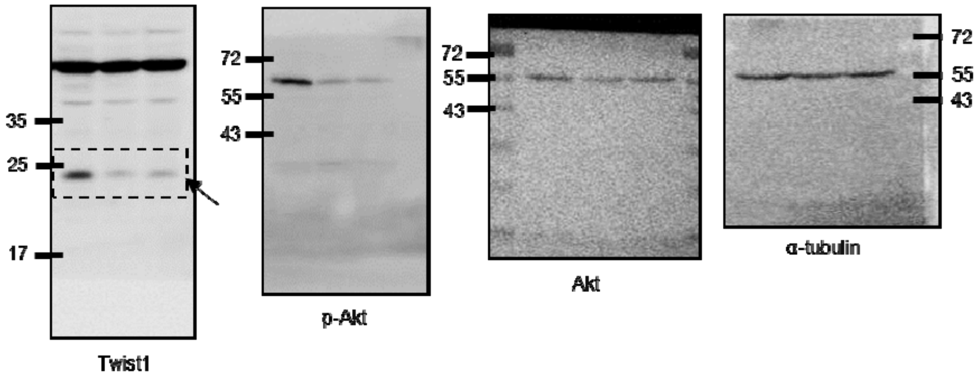

Supplementary Figure S9B

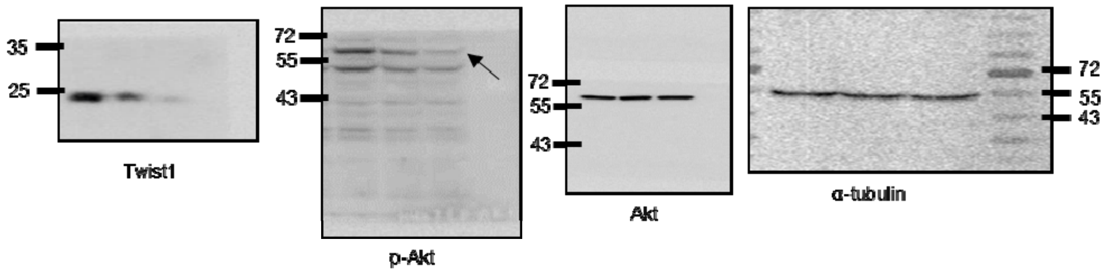

Supplementary Figure S10A

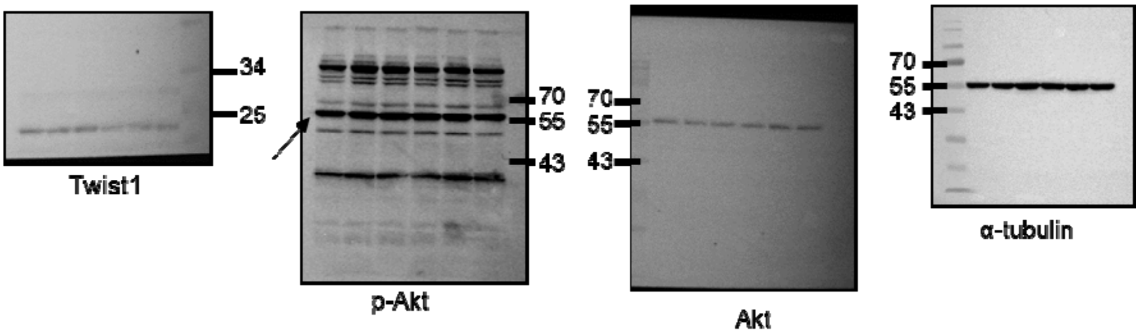

Supplementary Figure S10B

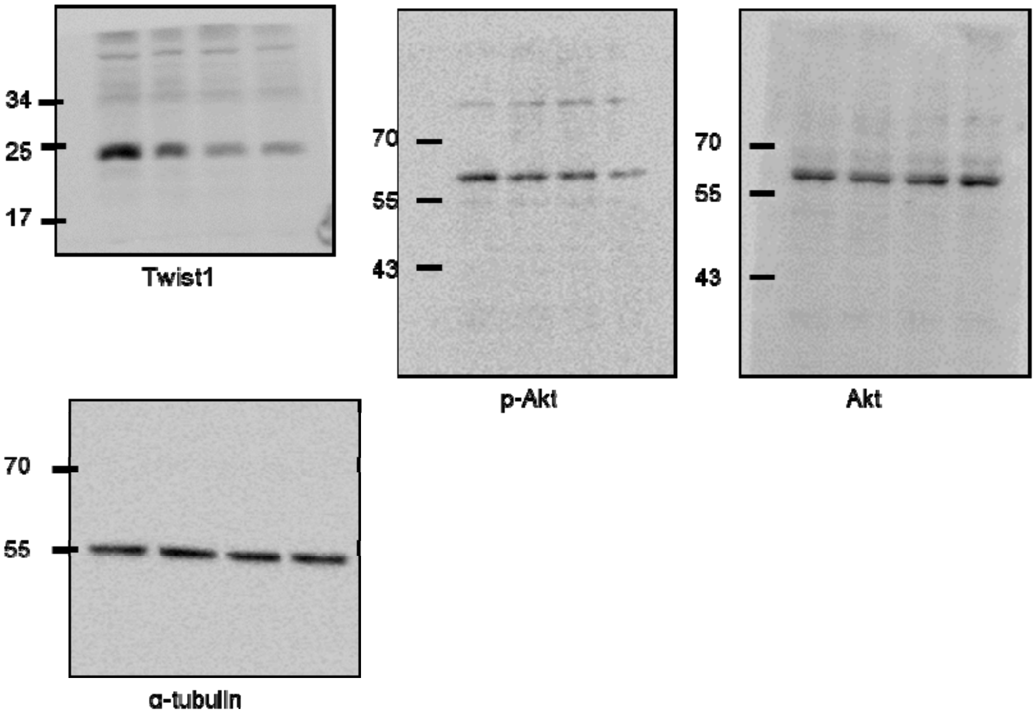

Supplementary Figure S11

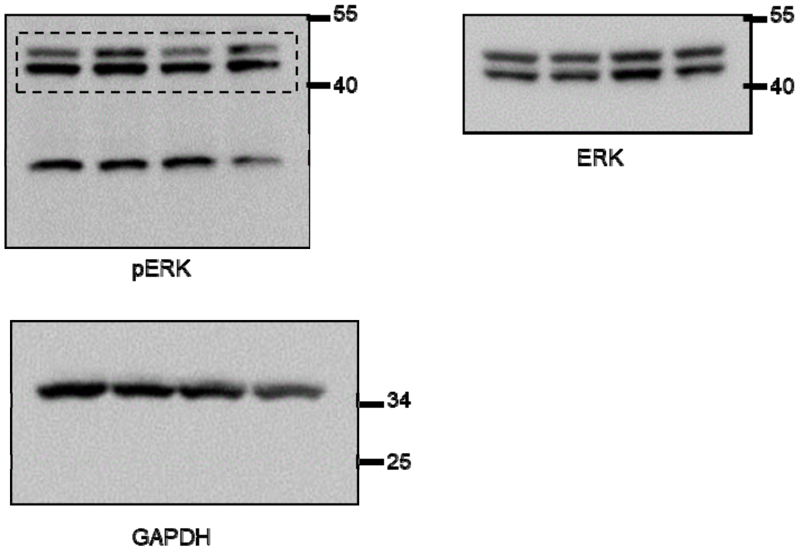

Supplementary Figure S12A

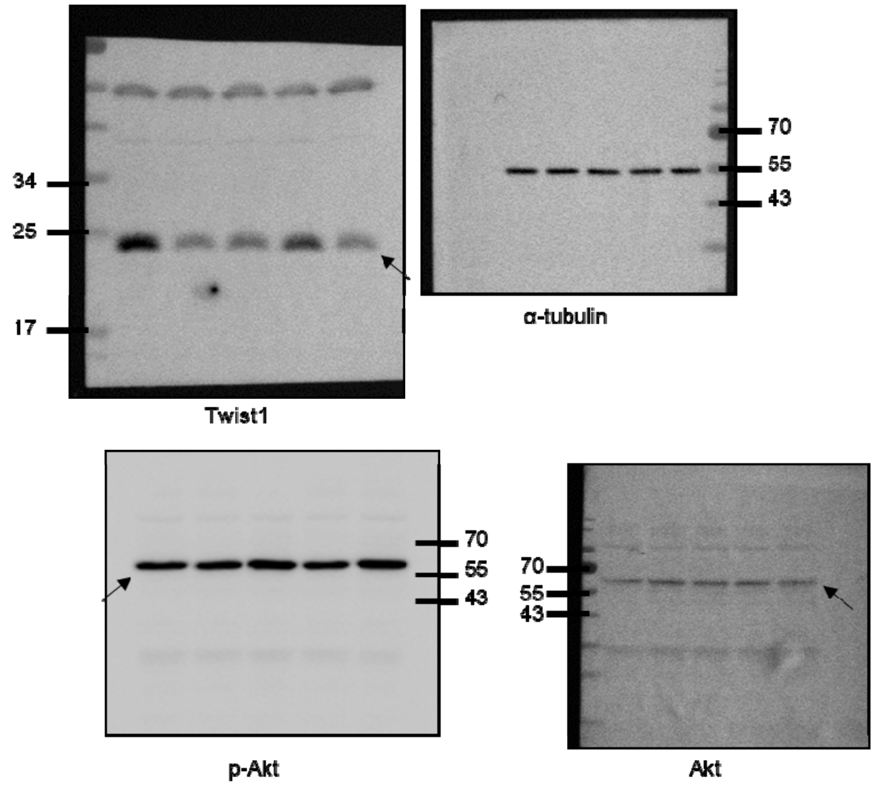

Supplement: Supplementary file 1 — Supplementary Information. [file 41598_2021_95933_MOESM1_ESM.pdf]
